# JustOrthologs: a fast, accurate and user-friendly ortholog identification algorithm

**DOI:** 10.1093/bioinformatics/bty669

**Published:** 2018-08-01

**Authors:** Justin B Miller, Brandon D Pickett, Perry G Ridge

**Affiliations:** Department of Biology, Brigham Young University, Provo, UT, USA

## Abstract

**Motivation:**

Orthologous gene identification is fundamental to all aspects of biology. For example, ortholog identification between species can provide functional insights for genes of unknown function and is a necessary step in phylogenetic inference. Currently, most ortholog identification algorithms require all-versus-all BLAST comparisons, which are time-consuming and memory intensive.

**Results:**

In contrast to existing approaches, JustOrthologs exploits the conservation of gene structure by using the lengths of coding sequence regions and dinucleotide percentages to identify orthologs. In comparison to OrthoMCL, OMA and OrthoFinder, JustOrthologs decreases ortholog identification runtime by more than 96% and achieves comparable precision and recall scores. The computational speedup allowed us to conduct pairwise comparisons of 1197 complete genomes (780 eukaryotes and 417 archaea). We confirmed gene annotations for 384 120 genes, grouped 1 675 415 genes in previously unreported ortholog groups, and identified 51 429 potentially mislabeled genes across 622 843 ortholog groups.

**Availability and implementation:**

JustOrthologs is an open source collaborative software package available in the GitHub repository: https://github.com/ridgelab/JustOrthologs/. All test FASTA files used for comparisons are freely available at https://github.com/ridgelab/JustOrthologs/comparisonFastaFiles/. Reference genomes used in this work are available for download from the NCBI repository: ftp://ftp.ncbi.nih.gov/genomes/.

**Supplementary information:**

Supplementary data are available at *Bioinformatics* online.

## 1 Introduction

Ortholog identification has long been a daunting, yet critical, first step for many studies. Orthologs are gene sequences derived from the same ancestral gene present in two species’ last common ancestor, and can provide support in phylogenetic tree reconstruction or insights into gene function (Koonin, 2005).

Unsurprisingly, many ortholog identification algorithms are currently available. Unfortunately, existing algorithms are complex and hampered by poor performance. OrthoMCL requires a complicated 13-step process, which involves an all-versus-all BLAST comparison, a Markov Clustering (MCL) algorithm, and construction of a MySQL database to identify ortholog groups (Li *et al.*, 2003). OrthAgogue attempts to simplify the process by combining the MCL into a single step, and decreases the number of steps required in an OrthoMCL analysis from 13 to 8 (Ekseth *et al.*, 2014); however, the eight-step process is still overwhelming for the average biologist. Using a different approach, OrthoFinder increases ortholog precision by taking into account a gene length bias associated with the all-versus-all BLAST scores (Emms and Kelly, 2015). While OrthoFinder is a single-step process, it still requires the installation of several software dependencies and is time-consuming to run. OMA evaluates the evolutionary relationships between proteomes through a pairwise comparison, with additional web interfaces and tools for querying their databases (Altenhoff *et al.*, 2015). OMA has over a dozen major releases, each of which increased the number of proteomes in the database. However, it requires a strict directory structure for independent ortholog identification and is not easily scriptable. Other algorithms, such as Inparanoid (Sonnhammer and Östlund, 2015), EggNOG (Huerta-Cepas *et al.*, 2016), OrthoDB (Zdobnov *et al.*, 2017) and TreeFam (Schreiber *et al.*, 2014) take a similar approach to OMA by maintaining a database of orthologous groups and providing tools to BLAST a query sequence against their respective database. While each software package implements a slightly different ortholog identification algorithm, each method is based on time-intensive all-versus-all BLAST comparisons for the initial scoring, which limits the typical dataset to a few specific genes of interest. Furthermore, external dependencies, intricate step-by-step processes or a strict directory structure are often required, precluding inexperienced researchers from using these programs to identify orthologs. Therefore, comprehensive comparisons between algorithms require not only an analysis of accuracy, but also an evaluation of runtime complexity and ease of user experience. A comparison of the strengths and weaknesses of each of the three algorithms used for comparisons is found in Supplementary Table S1.

JustOrthologs is unlike any other ortholog identification algorithm. It exploits the conservation of coding sequence (CDS) region length to reduce the number of gene–gene sequence comparisons. By sorting each FASTA file by the number of CDS regions in each gene (i.e. the number of coding exons), fewer direct comparisons are required. Furthermore, rather than compare whole sequences (i.e. a BLAST comparison), JustOrthologs compares dinucleotide percentages to determine the level of sequence identity between two CDS regions. These innovations reduce runtime by at least 96% compared with other popular ortholog identification algorithms. Moreover, JustOrthologs has no external dependencies, has only a few, well-documented parameters, and requires only a single step at runtime.

## 2 Materials and methods

### 2.1 Algorithm design

Although JustOrthologs is run by a single command, the algorithm implements a two-step process. First, JustOrthologs utilizes a previously unreported conservation in CDS region length within orthologs. JustOrthologs compares CDS region lengths and requires that the two genes, with a couple exceptions, have CDS regions of the exact same lengths. JustOrthologs allows up to two CDS regions to differ in length within each sequence, thereby accommodating exon fusion and splitting events. Furthermore, since genes are sorted by the number of CDS regions, and only two fusion or splitting events are allowed, if the difference between the number of CDS regions in the query and subject sequences exceeds two, the remaining genes in the file are not compared. By limiting comparisons to only CDS regions, as described above, we significantly decrease the number of pairwise comparisons between genes.

Second, we further reduce computational complexity by completely avoiding BLAST comparisons in favor of dinucleotide usage percentages. A dinucleotide percentage is calculated by counting the occurrences of a dinucleotide pair in an exon and dividing by the total number of dinucleotide pairs in that exon. This process is repeated for each of the 16 possible combinations of dinucleotides (e.g. AG, CT, CC, etc.), and then repeated for each exon, creating dinucleotide motifs that can be compared between exons in other genes. If the difference in dinucleotide percentages between two sequences is lower than a threshold, and the lowest among possible orthologs in the subject file, then that gene is reported as orthologous to the query. Nucleotide bigrams were used to allow for greater sequence divergence within each CDS region, especially at the third codon position. See Supplementary Figure S1 for an outline of the decision process for JustOrthologs.

We present three settings for JustOrthologs, each refined for a specific case: (i) comparison of closely related species, (ii) comparison of distantly related species, and (iii) a combination of the first two options to report the highest number of orthologs. Pseudocode for each of the three settings can be found in Supplementary Algorithms 1, 2 and 3, respectively.

Thresholds for dinucleotide percentages are set depending on which of the three use cases, described above, is set. For closely related species, the recommended threshold is 0.05, while distantly related species have a recommended threshold of 0.1. Both thresholds were tuned and calculated using species not shown in this paper so as not to inadvertently train our thresholds on our test cases. We tuned the threshold for closely related species by examining the precision and accuracy of recovered orthologs between *Alligator sinensis* and *Alligator mississippiensis* [52 MYA estimated time of divergence (Hedges *et al.*, 2006, 2015; Kumar and Hedges, 2011; Kumar *et al.*, 2017)] and *Myotis lucifugus* and *Myotis brandtii* [14.2 MYA estimated time of divergence (Hedges *et al.*, 2006, 2015; Kumar and Hedges, 2011; Kumar *et al.*, 2017)] for thresholds between 0.01 and 1.00, incremented by 0.01. The same process was completed for orthologs recovered from the more distantly related species, *Alligator sinensis* and *Myotis lucifugus* [312 MYA estimated time of divergence (Hedges *et al.*, 2006, 2015; Kumar and Hedges, 2011; Kumar *et al.*, 2017)]. The threshold score is adjustable (see Supplementary Note for description on how to tune these thresholds using other species), although we have provided recommended thresholds based on our analyses.

All three settings of JustOrthologs are parallelized with the default setting to use as many cores as the system has available. Alternatively, the user may specify the number of cores. To improve the user experience, intuitive, well-documented argument parsing is included. A provided wrapper script allows users to extract all ortholog pairs from two FASTA files and two General Feature Format 3 (GFF3) files with options to extract all CDS regions, to sort based on the number of CDS regions, to filter based on gene annotation, and then to run any version of JustOrthologs and find all ortholog pairs between the two species. We provide a comprehensive README and README_WRAPPER for argument descriptions, as well as example FASTA and GFF3 files in the GitHub repository.

### 2.2 Ortholog identification across 1197 species

A common practice is to find orthologous genes across a group of species. Since JustOrthologs is designed for pairwise species comparisons, an independent Python script (combineOrthoGroups.py with accompanying documentation in README_OTHER_PROGRAMS) was written to combine the output from multiple JustOrthologs output files. CombineOrthoGroups takes as input a directory with the output files from one or more species comparisons completed using JustOrthologs. It reads each file, adding the pairwise ortholog groups to a dictionary of all ortholog pairs. It then finds all genes that belong to a group (e.g. if gene A in species 1 points to gene B in species 2 and gene B in species 2 points to gene C in species 3, then the ortholog group would contain genes A, B and C). Because we are interested in identifying potentially mislabeled or previously unidentified orthologs, we applied a filter which requires one-to-one orthology (i.e. two genes from the same species cannot be reported as orthologous). While we realize that one-to-one orthology is not always the best representation of phylogenetic history due to gene duplication, horizontal gene transfer, etc., one-to-one orthology ensures that orthologs are grouped based on the most-probable orthology and not because of paralogy or software error.

### 2.3 Generating test data

Since JustOrthologs requires DNA sequences and CDS annotations, we were unable to use traditional ortholog data sets (e.g., such as OrthoBench (Trachana *et al.*, 2011)), which contain protein sequences without splice site annotations. Therefore, we relied on the Human Genome Organization Gene Nomenclature Committee (HGNC) gene annotations and outline the creation of test data sets in Supplementary Figure S2. The HGNC uses ortholog annotations established by SWISS-PROT and the HGNC interacts with various nomenclature groups to ensure that orthologous genes between different species are assigned the same symbol. All FASTA sequence data for our main comparisons between 1197 genomes and our pairwise comparisons between *Homo sapiens*, *Pan paniscus*, *Falco peregrinus* and *Equus caballus*, were downloaded and extracted from the reference genomes and GFF3 files found in the NCBI database in September, 2017 (Pruitt *et al.*, 2014; Tatusova *et al.*, 2014). All 1197 species are listed in Supplementary Table S2.

Three types of test data sets were created, each outlined in Supplementary Figure S2: (i) original, in which all genes included from species 1 have their true ortholog in species 2 included in the test set [i.e. everything in these test sets are true positives (TPs)], (ii) mismatch, which contains a mix of genes and their true orthologs, and genes with no orthologs in the data set—these test sets most closely approximate an unfiltered data set that might be used in research because they have a mix of TPs and false positives (FPs) and (iii) error, which contain no TP orthologs (i.e. any orthologs identified in these test sets are FPs). Each test set includes up to 1000 genes. Once a test set has 1000 genes, a new test set is created starting where the last test set left off. In our test sets, mismatch test sets had 50–90% TPs. This process resulted in 33 test sets (11 of each type) for human versus falcon, 39 test sets (13 of each type) for human versus horse and 45 test sets (15 of each type) for human versus bonobo.

We report estimated species divergence times between *Homo sapiens* (GCF_000001405.28), *Pan paniscus* (GCF_000258655.2), *Falco peregrinus* (GCF_000337955.2) and *Equus caballus* (GCF_000002305.2) in Table 1 to show that our comparisons span both closely and distantly related species. Filters were applied to these data to remove annotated translational errors, suspected errors and unclassified transcription discrepancies. Similar to previous studies (Camiolo *et al.*, 2015), we included only the longest isoform of each gene in our analyses. To generate our test data, we relied on an upper and lower case insensitive review of gene names that were annotated by the HGNC (Gray *et al.*, 2015) to divide genes into several groups for testing as described below. Orthologs were considered TP if they matched the HGNC annotations, FP if they did not match HGNC annotations, and false negatives (FN) if genes with matching HGNC annotations were not reported. Any orthologs reported for the error data set were by definition FPs, as no TPs were possible.

**Table 1.** Estimated time of species divergence

Species 1	Species 2	Estimated time	Median time	Confidence interval
*Homo sapiens*	*Pan paniscus*	6.65 MYA	6.4 MYA	6.23–7.07 MYA
*Homo sapiens*	*Equus caballus*	96 MYA	94 MYA	91–102 MYA
*Homo sapiens*	*Falco peregrinus*	312 MYA	320 MYA	297–326 MYA
*Pan paniscus*	*Equus caballus*	96 MYA	94 MYA	91–102 MYA
*Pan paniscus*	*Falco peregrinus*	312 MYA	320 MYA	297–326 MYA
*Equus caballus*	*Falco peregrinus*	312 MYA	320 MYA	297–326 MYA

*Note*: Species Divergence taken from the average estimate from various studies included in TimeTree (Hedges *et al.*, 2006, 2015; Kumar and Hedges, 2011; Kumar *et al.*, 2017).

We recognize that some HGNC gene annotations are potentially incorrect. However, these annotations are reliable for our testing and algorithm comparisons for two reasons. First, it is likely that a large majority of the annotations are correct, and since we use a total of 51 721 genes between the four species for testing, a small fraction of incorrect labels is unlikely to significantly affect the results. Second, all algorithms were evaluated using the same data sets, so all algorithms are subject to the same potentially incorrect annotations present in the test data sets.

### 2.4 Comparisons to OrthoMCL, OMA and OrthoFinder

The OrthoMCL pipeline (Li *et al.*, 2003) has many steps and can be difficult to use. Nevertheless, the process is relatively well-documented. During the all-versus-all BLAST, we used the NCBI BLAST+ suite version 2.2.28 (Camacho *et al.*, 2009) instead of the legacy BLAST suite (Altschul *et al.*, 1990). We used the BLAST+ provided Perl script, legacy_blast.pl, to convert the BLAST command to the correct form for BLAST+. Further modifications were required to obtain the desired output because the provided script is intended only as a starting point. After carefully reading the BLAST+ documentation, parameters for the final BLAST+ command were: -evalue 1e-5 -seq ‘yes’ -num_descriptions 10 000 -soft_masking true -outfmt 6. All other commands for OrthoMCL were as outlined in the original manuscript (Li *et al.*, 2003) and the step-by-step processes for OMA (Altenhoff *et al.*, 2015) and OrthoFinder (Emms and Kelly, 2015) were executed without modification.

### 2.5 Performance measurements

Similar to the method outlined by Emms *et al.* (Emms and Kelly, 2015), we used precision and recall to evaluate our algorithms. In our study, precision is the ratio of TP orthologs reported to total orthologs reported, while recall is the ratio of TP orthologs reported to all possible real orthologs in each data set:

$$Precision=\frac{TP}{TP+FP},\;Recall=\frac{TP}{TP+FN}$$

Some algorithms that we compared also searched for orthologs within the same species. JustOrthologs does not have this functionality, due to the high similarity of isoforms within a species and the rarity of such orthologs. Therefore, to ensure a fair comparison between algorithms, if an algorithm reported orthologs that did not include a sequence from each of the two species being compared, those specific orthologs were excluded from evaluation (e.g. in the *Homo sapiens* and *Pan paniscus* comparison, one gene from each of the species is required, as opposed to both genes being from a single species). Remaining groups were considered TPs if the groups had exactly one sequence from each species (as opposed to two or more from one or both species) and the gene names matched (i.e. the group exhibited one-to-one orthology between the two species). All tests were performed on an Intel Haswell (2.3 GHz) node with 24 cores. We allocated one node and 16 cores to each algorithm.

## 3 Results

### 3.1 Comparisons

#### 3.1.1 Precision

Precision evaluates the confidence that ortholog pairs are correct. JustOrthologs had the best precision of the algorithms tested, with nearly 100% precision for each test data set. OrthoFinder also had 100% precision for all test sets, except human versus falcon, for which no ortholog pairs were reported. OrthoMCL had the lowest precision (∼55–80%) for all test sets, while OMA had high precision (∼100%) when only orthologs are present, but lower precision (∼96%) when mismatches are present in the test data (Supplementary Figs S3 and S4).

#### 3.1.2 Recall

Recall measures the number of correctly reported ortholog pairs out of the number of possible real ortholog pairs. JustOrthologs, OMA and OrthoMCL had nearly 100% recall for human versus bonobo. For all three test sets, recall for JustOrthologs was much higher than OrthoFinder. Recall for JustOrthologs is comparable with the recall from OrthoMCL and OMA for closely related species, but JustOrthologs’ recall was significantly lower for more distantly related species (Supplementary Figs S5 and S6). As expected from the algorithm’s implementation, recall for JustOrthologs increases when more CDS regions are present in a gene because significant mutations within a few CDS regions can indicate speciation events while the remaining CDS regions remain relatively unchanged.

#### 3.1.3 False positive rate

We used the error data sets to assess TP rates. OrthoFinder did not report any TPs in any of the data sets. Likewise, JustOrthologs reported no TPs for human versus bonobo and human versus falcon test cases, but had a TP rate of 0.008% for the human versus horse test cases. All other algorithms had high TP rates: OrthoMCL (27–42%) and OMA (11–12.5%) (Supplementary Figs S7 and S8).

#### 3.1.4 Performance

Since all-versus-all BLAST requires comparing all sequences within the same file (once for each file), and all sequences between files (using each file once as the subject), big-O time complexity for ortholog pair identification using all-versus-all BLAST based algorithms (i.e. all algorithms except JustOrthologs) is typically O(*n*^4^), where *n* is the number of sequences analyzed. In contrast, the time complexity of JustOrthologs is a function of the number of genes with similar numbers of CDS regions (c) and the lengths of the compared CDS regions (l). Both values are usually significantly smaller than the total number of genes or the total number of CDS regions, and have very small constant factors. For the dinucleotide percentages that are actually compared, they are compared in a pairwise manner, leaving the maximum time complexity as O(c^2^l^2^). In real-world scenarios, where relatively few genes contain similar numbers of CDS regions, the time complexity is more similar to a logarithmic function because the initial sorting step limits sequence comparisons to only sequences with similar numbers of CDS regions. The dinucleotide comparisons also reduce complexity because the actual sequences are never aligned. The third setting of JustOrthologs, which is a combination of the first two, is twice as computationally intensive [O(2c^2^l^2^)] because it requires running both algorithms before combining the output from each.

We compared the user time, which accounts for execution time of each thread, (i.e. JustOrthologs gained no advantage in this comparison by having more efficient multi-threading) for each of the algorithms across all test data sets. JustOrthologs was substantially faster than all other algorithms, even in its slowest setting. The slowest setting of JustOrthologs was on average 28× faster than OrthoMCL, 96× faster than OMA and 4900× faster than OrthoFinder. The two faster settings of JustOrthologs were always at least 58× faster than all other algorithms (Supplementary Fig. S9).

Furthermore, the multiprocessing capabilities of JustOrthologs surpass all other algorithms, with an average core utilization of 11.3 out of the 16 allocated cores. In comparison, OMA averaged ∼1.25 cores, OrthoFinder averaged ∼5.0 cores and OrthoMCL averaged ∼6.6 cores out of 16 allocated cores, thus when comparing the use of each algorithm in a realistic setting (i.e. multi-threaded), JustOrthologs provides a more substantial advantage than reported here.

### 3.2 Results for individual tests

Precision, recall and user time for individual tests for each algorithm are found in Supplementary Figures S10–S33.

### 3.3 Ortholog identification in 1197 species

Finally, as proof-of-concept, we used JustOrthologs to perform a pairwise comparison of all genes in 1197 species. JustOrthologs finished each genome-wide pairwise comparison in 0–24 h, depending on the number/length of annotated genes. In total, all pairwise comparisons took 45 476 h to complete. We identified 1 675 415 currently unnamed genes that were classified as orthologous to other genes in different species. We also identified 51 429 potentially mislabeled genes, which we report. We report the first 30 ortholog groups identified by JustOrthologs in Table 2 and examine potentially mislabeled genes within those groups. In Table 2, several ortholog groups have poor sequence alignments. In Supplementary Note S2, we explain why a poor alignment might occur and give an example of two simulated sequences with a poor alignment that would be identified as orthologous. We have included a comprehensive list of all orthologous gene groups identified in these comparisons in Supplementary Table S3. Supplementary Tables S4 and S5 analyze the composition of these groups by reporting the annotations and group sizes, respectively. We propose that the annotations of each of these genes should be examined and updated by the HGNC.

**Table 2.** Ortholog groups recovered using JustOrthologs and CombineOrthoGroups

Genes with the same annotation	Genes with other annotations	Genes with unknown annotations	Total genes	Reason for other annotations
127	0	63	190	N/A
178	0	7	185	N/A
172	1	7	180	XP_018109801.1 has 100% BLAST identity with NP_001087532.1, which is annotated the same as the other 172 genes
155	2	21	178	The nucleotide composition and exon length of XP_001959559.1 and XP_002071834.1 are similar to XP_010179458.1. However, the alignment is very different. These two genes are probably incorrectly reported as orthologous by JustOrthologs
169	0	9	178	N/A
169	1	5	175	XP_414807.2 has a 99% BLAST identity with XP_015732072.1 from a closely related species, which is annotated the same as the other 169 genes
166	0	5	171	N/A
165	1	5	171	NP_068697.1 is annotated Trp53inp1 instead of TP53INP1
163	1	6	170	XP_014347657.1 is annotated LRRC8E instead of LRRC8C
165	0	4	169	N/A
161	0	7	168	N/A
162	0	5	167	N/A
161	1	4	166	XP_020368157.1 is incorrectly reported as orthologous by JustOrthologs. The CDS region lengths matched some exons in XP_005866852.1, but the alignment of the sequences was very poor
163	0	3	166	N/A
152	1	13	166	XP_018123052.1 is annotated grb10.L instead of GRB10
161	0	4	165	N/A
156	0	9	165	N/A
159	0	6	165	N/A
160	0	5	165	N/A
160	0	4	164	N/A
159	0	5	164	N/A
158	0	5	163	N/A
156	1	5	162	XP_017312051.1 is incorrectly reported as orthologous by JustOrthologs. The CDS region lengths matched several exons within XP_020920808.1, but the alignment of the sequences was poor
156	0	5	161	N/A
158	0	3	161	N/A
153	0	7	160	N/A
149	0	9	158	N/A
154	0	3	157	N/A
146	0	11	157	N/A
153	0	4	157	N/A

*Note*: The first 30 ortholog groups are ordered from the most genes to the fewest genes. The first column shows the number of genes with the same annotations. The second column shows the number of genes with a different annotation than the genes in the first column. The third column shows the number of genes without annotations. The fourth column shows the total number of genes in the ortholog group. The fifth column is an analysis of why genes in the second column were not annotated the same as genes in the first column but were reported as orthologous by JustOrthologs. Each gene comes from a different species.

All ortholog identification algorithms are limited by their ability to successfully differentiate between paralogs and orthologs. Therefore, individual species comparisons where whole genome duplications occurred or where many homologs exist generally cause algorithms to report a higher number of TP orthologs. In our comparison of 1197 species, we also analyzed specific pairwise gene comparisons. We show 15 pairwise comparisons of complete genomes across diverse taxa in Table 3. We did not subsample genes from these data, which allows of a more complete view of how JustOrthologs performs on real-world datasets. Although recall is significantly affected in some species comparisons, JustOrthologs maintains high precision in all instances. Furthermore, thousands of previously unnamed genes are identified in orthologous pairs, facilitating the evaluation of their orthologous relationship. In the aforementioned orthology groups, we performed a strict one-to-one orthology filter to combine these pairwise relationships to minimize compounding TP relationships.

**Table 3.** Whole genome comparison of different species

Species 1	Species 2	Number of genes in species 1	Number of genes in species 2	Number of shared ortholog annotations from HGNC	True positives reported	False positives reported	Unnamed genes reported in orthologous pairs	Precision (%)	Recall (%)
*Homo sapiens*	*Pan paniscus*	20 088	17 900	14 653	14 119	462	905	96.83	96.36
*Homo sapiens*	*Equus caballus*	20 088	16 691	12 725	8229	150	246	98.21	64.67
*Homo sapiens*	*Falco peregrinus*	20 088	12 643	10 659	841	38	35	95.68	7.89
*Gallus gallus*	*Falco peregrinus*	16 420	12 643	9163	5132	139	597	97.36	56.01
*Astyanax mexicanus*	*Danio rerio*	21 920	22 408	5832	683	296	688	69.77	11.71
*Cynoglossus semilaevis*	*Danio rerio*	19 450	22 408	5699	199	104	205	65.68	3.49
*Oncorhynchus kisutch*	*Salmo salar*	30 680	40 642	2800	2424	183	18 300	92.98	86.57
*Oreochromis niloticus*	*Pundamilia nyererei*	27 785	21 832	8645	8326	94	9857	98.88	96.31
*Alligator mississippiensis*	*Crocodylus porosus*	17 492	13 837	10993	10 238	4	1615	99.96	93.13
*Mus musculus*	*Rattus norvegicus*	21 815	21 481	15199	12 183	720	279	94.42	80.16
*Bos taurus*	*Capra hircus*	17 980	19 208	12894	11 929	97	1337	99.19	92.52
*Bos taurus*	*Vicugna pacos*	17 980	16 297	11411	7991	18	502	99.78	70.03
*Calypte anna*	*Haliaeetus leucocephalus*	12 225	14 150	9825	7041	15	662	99.79	71.66
*Calypte anna*	*Chaetura pelagica*	12 225	11 852	8770	6565	14	695	99.79	74.86
*Prunus avium*	*Prunus mume*	24 179	22 628	0	0	0	14 004	N/A	N/A

*Note*: All available genes are compared between various species. The first two columns are the names of the species being compared. Columns three and four indicate how many genes are present in each species. Column five shows how many genes have the same ortholog annotations in both species. Column six shows the number of true positives JustOrthologs identifies. Column seven shows the number of false positives identified by JustOrthologs. Column eight shows the number of genes reported as orthologous by JustOrthologs but not named by the HGNC. Columns nine and ten report the precision and recall of the compared species, respectively.

## 4 Discussion

JustOrthologs significantly decreases ortholog classification runtimes, allowing faster ortholog comparisons on larger gene data sets than any other ortholog identification algorithm. The higher precision of JustOrthologs offers users more confidence in ortholog pairs identified by JustOrthologs than orthologs identified by OrthoMCL or OMA. JustOrthologs also offers higher recall in genes from closely related species with many CDS regions than any other algorithm, allowing better identification of orthologs with many splice sites. As might be expected, all ortholog identification algorithms perform best when analyzing closely related species such as *Homo sapiens* versus *Pan paniscus*. Compared to other algorithms, JustOrthologs had a higher combined precision and recall score than any other algorithm for all test sets for closely related species. In more distantly related species, such as *Homo sapiens* versus *Equus caballus*, only OrthoFinder was more precise than JustOrthologs, but OrthoFinder had much lower recall—JustOrthologs identified over 6000 ortholog groups that OrthoFinder missed. For more distantly related species, such as *Homo sapiens* versus *Falco peregrinus*, OrthoFinder reported no ortholog pairs, but JustOrthologs reported over 1000 ortholog pairs, while maintaining ∼99% precision. In contrast, less precise methods, such as OrthoMCL, reported only 70–80% precision on the same data sets. Overall, JustOrthologs is the most consistent performer among tested algorithms, and is significantly faster.

The decreased runtime allows JustOrthologs to perform whole genome analyses of diverse species that were previously impossible to perform. Since JustOrthologs uses a unique algorithm that does not rely on time-consuming all-versus-all BAST comparisons, it enables researchers to quickly identify potential orthologs using whole genome analyses. Since we opted to have higher precision than recall, orthologs reported by JustOrthologs have high precision, which allows researchers to have confidence in the reported ortholog pairs.

Moreover, JustOrthologs has comprehensive documentation and, compared to other algorithms, is easy to use. These characteristics, the provided wrapper scripts, and the single-step command line process that does not require any external software, make JustOrthologs accessible to even individuals with limited programing experience.

Although JustOrthologs is a novel approach that accurately and precisely recovers orthologous gene relationships without a sequence alignment, a sequence alignment could be used to evaluate proposed orthologous relationships identified by JustOrthologs. Since all-versus-all BLAST searches are computationally intractable when the number of sequences is large (e.g. whole genome analyses), using BLAST to evaluate the sequence alignments of the proposed orthologous pairs could be used to further improve accuracy with a limited computational cost. However, we opted not to include an alignment step in our algorithm to illustrate the predictive power of our novel approach. Furthermore, our approach allows for structural variants and rearrangements that a sequence alignment might miss.

Since JustOrthologs exploits CDS region length conservation, the algorithm works only with annotated CDS. However, as whole genome and transcriptome sequencing is becoming increasingly common, owing to reduced prices and better assembly/annotation software, this limitation is likely to decrease with time. Furthermore, JustOrthologs is better suited than any existing algorithm to handle the large data sets that have become the norm in biology. As evidence of the potential utility of JustOrthologs, we identified orthologous groups within 1197 species, in 45 000 h of real time using 16 processing cores (we farmed the analysis out to multiple processing nodes, so real time was calculated by summing the real time from each of the nodes). Extrapolating from measured times, such a comparison would not have been possible for any of the other algorithms compared in this manuscript.

The gold standard in science is perfectly accurate and complete data; however, few algorithms are capable of delivering both. We deliberately opted for JustOrthologs to have higher precision than recall, because as biologists we prioritize confidence in the accuracy of our data as opposed to being comprehensive. For closely related species, the tradeoff is almost unnoticeable. However, similar to OrthoFinder, greater evolutionary distance between genes significantly decreases the recall of JustOrthologs. Nevertheless, recall for JustOrthologs significantly outperforms OrthoFinder for distantly related species.

JustOrthologs is a unique algorithm for ortholog identification as it departs from the traditional all-versus-all BLAST search algorithms that have saturated ortholog identification for the past decade. While all-versus-all BLAST has proven useful for small-scale analyses, its O(n^4^) runtime is prohibitive for species-wide ortholog identification. In fact, two algorithms, OMA and OrthoFinder, are incapable of completing a genome-wide ortholog comparison in a week. In an era of high throughput sequencing, an algorithm capable of efficiently searching entire genomes is necessary.

## Supplementary Material

Supplementary Data
