# Supplementary material for: JustOrthologs: a fast, accurate and user-friendly ortholog identification algorithm
Source: Bioinformatics. 2018 Aug 1;35(4):546–52. doi: 10.1093/bioinformatics/bty669 (PMC6378933; doi:10.1093/bioinformatics/bty669)
Supplement: Supplementary Data [file bty669_supp.zip › bty669-suppl_data/JustOrthologs Supplement.pdf]

JustOrthologs Supplementary Information

Algorithms

Supplementary Algorithm 1:

Algorithm 1: JustOrthologs

```
function compareSequenceSets(f1, f2):
Input: 2 sorted sets of sequences
Output: set of orthologous pairs
orthologous_pairs = {}
for s1 in f1:
    best_total = 2 * (getCdsCount(s1) - 1)
    overall_best_seq = ''
    for s2 in f2:
        total = 0
        bssf = 0.05
        best_seq = ''
        if |getCdsCount(s1) – getCdsCount(s2)| <= 3:
            for c1 in s1:
                for c2 in s2:
                    if |c1| == |c2| && |c2| > 15:
                        d = sumDifferencesOfAllDinucleotideCounts(c1, c2)
                        if d < bssf:
                            bssf = d
                            best_seq = s2
                if bssf < 0.05: total += bssf
                else: total += 2
            if type(total) == "float" && total <= best_total:
                best_total = total
                overall_best_seq = best_seq
    if |overall_best_seq| > 0:
        orthologous_pairs ∪= {(s1, overall_best_seq)}
return orthologous_pairs

Input: 2 sets of sequences as f1, f2
Output: orthologs
orthologs = compareSequenceSets(sort(f1), sort(f2)) ∪= compareSequenceSets(sort(f2), sort(f1))
for o in orthologs:
    if !( oi-1 -> oi-2 && oi-2 -> o-j):
      print o
```

Supplementary Algorithm 2:

Algorithm 2: JustOrthologs -d

```
function compareSequenceSets(f1, f2):
Input: 2 sorted sets of sequences
Output: set of orthologous pairs
orthologous_pairs = {}
for s1 in f1:
    overall_best_seq = ''
    for s2 in f2:
        total = 0
        bssf = 0.1
        best_seq = ''
        if |getCdsCount(s1) – getCdsCount(s2)| <= 3:
            for c1 in s1:
                for c2 in s2:
                    if |c1| == |c2| && |c2| > 15:
                        d = sumDifferencesOfAllDinucleotideCounts(c1, c2) – 2.2
                        h = highestDinucleotideDifference(c1, c2)
                        if h < 0.03 && d < bssf:
                            bssf = d
                            best_seq = s2
                if bssf < 0.1: total += bssf
                else: total += 2
            if type(total) == "float" && ( (getCdsCount(s1) < 6 && total < -2.15) || (getCdsCount(s1) >= 6 && total < 0) ):
                best_total = total
                overall_best_seq = best_seq
    if |overall_best_seq| > 0:
        orthologous_pairs ∪= {(s1, overall_best_seq)}
return orthologous_pairs

Input: 2 sets of sequences as f1, f2
Output: orthologs
orthologs = compareSequenceSets(sort(f1), sort(f2)) ∪= compareSequenceSets(sort(f2), sort(f1))
for o in orthologs:
    if !( oi-1 -> oi-2 && oi-2 -> o-j):
      print o
```

Supplementary Algorithm 3:

```
Algorithm 3: JustOrthologs -c

Input: 2 sets of sequences as f1, f2
Output: orthologs
orthologs = JustOrthologs(f1, f2) ∪= JustOrthologs –d (f1, f2)
for o in orthologs:
   if !( oi-1 -> oi-2 && oi-2 -> o-j):
      print o
```

Notes

Supplementary Note: Tuning the threshold parameter is a simple, yet time-consuming process. It requires orthologs to be annotated in the species used to tune the parameter. After choosing which species to tune the threshold parameter, run JustOrthologs for values between 0.01 and 1.00, incremented by 0.01 (i.e., 100 times), saving the output in different files. Then count the number of correctly classified orthologs, false positive orthologs, and calculate the precision and accuracy of each run. Based on precision and accuracy scores, choose the threshold value that best suites the needs of the research. The commands are presented for executing this process, where [s1] represents the first species, [s2] represents the second species, [o] represents the output file, and [t] represents the threshold.

JustOrthologs for closely related species:
`python justOrthologs.py -s [s1] -q [s2] -o [o] -r [t]`
JustOrthologs for distantly related species:
`python justOrthologs.py -d -s [s1] -q [s2] -o [o] -r [t]`
JustOrthologs combined approach:
`python justOrthologs.py -c -s [s1] -q [s2] -o [o] -r [t]`

Supplementary Note 2: Since JustOrthologs relies heavily on identifying CDS regions that are the same length, it is possible for two gene to have the same CDS region lengths and similar dinucleotide percentages without having similar sequences. For this reason, precision and accuracy are lowest when genes have fewer CDS regions. For instance, if a simulated gene has one CDS region with 21 nucleotides, it could randomly have very similar same dinucleotide percentage as a different sequence if portions of the gene are rearranged:

Sequence 1: ATGAAATTTCCCGGGATCTAA
Sequence 2: ATGCCCATCTTTAAAGGGTAA

In this case, the start codon and the stop codon are identical, but the sequence alignment would be very poor because of the nucleotide rearrangements. However, the sequences have the same nucleotide composition, and the same codons in a different order. So, two-thirds of the dinucleotide percentages will be identical even without chance collisions with subsequent codons (e.g ATCCAG and ATCCCC share a CC dinucleotide between codons).

Tables

Supplementary Table 1: Comparing the strengths and weaknesses of the algorithms used. All three algorithms also use a time-intensive all-versus-all BLAST to recover orthologous groups.

Algorithm	Advantages	Disadvantages
OrthoMCL	Widely used<br>High recall	Complicated 13-step process

OrthoFinder	Single-step process<br>High precision	Slow<br>Several software dependencies
OMA	Comprehensive ortholog database	Strict directory structure<br>Not easily scriptable

Supplementary Table 2: Species included in the combined analysis of 1 197 species. Please see "JustOrthologs Supplementary Table 2.csv"

Supplementary Table 3: A complete list of the ortholog groups identified by combineOrthoGroups. Please see "JustOrthologs Supplementary Table 3.csv" The first column is the gene name for the group with the most named genes. Each subsequent column includes a species name followed by a ":" followed by the gene accession number. If there is a gene annotation for that gene, it follows surrounded by parentheses. For example: "Parus_major:XP_015490149.1(KLHL23)" is a valid entry, where *Parus major* is the species name, XP_015490149.1 is the gene accession, and KLHL23 is the gene annotation.

Supplementary Table 4: Statistics for each ortholog group. Same as Table 2 from the main text, except all ortholog groups are included and the last column is not included. See "JustOrthologs Supplementary Table 4.csv"

Supplementary Table 5: A snapshot of how many genes are in each ortholog group. The first column is the number of genes in an ortholog group. The second column is the number of ortholog groups with that many genes in the group. See "JustOrthologs Supplementary Table 5.csv"

Figures

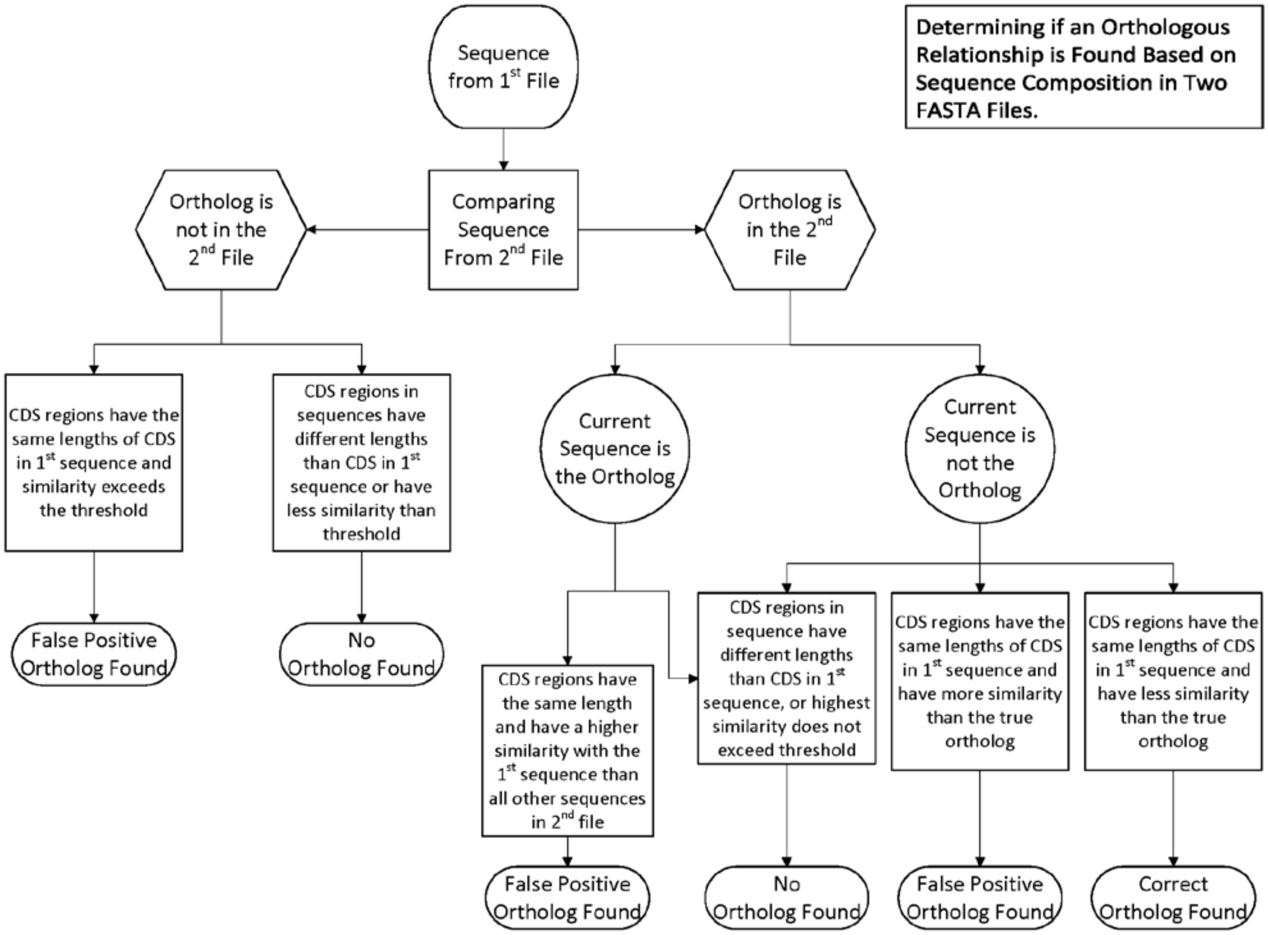

Supplementary Figure 1. JustOrthologs decision process. A query sequence is selected from the first file and is compared to one sequence at a time in the second file (subject file), and the processes JustOrthologs follows are outlined. The first sequence is from the first file (containing gene sequences from the first species), and the second sequence is from the second file (containing gene sequences from the second species). Details describing CDS comparison and dinucleotide percentage thresholds are described in the text. Similarity refers to the comparison of dinucleotide compositions of each exon.

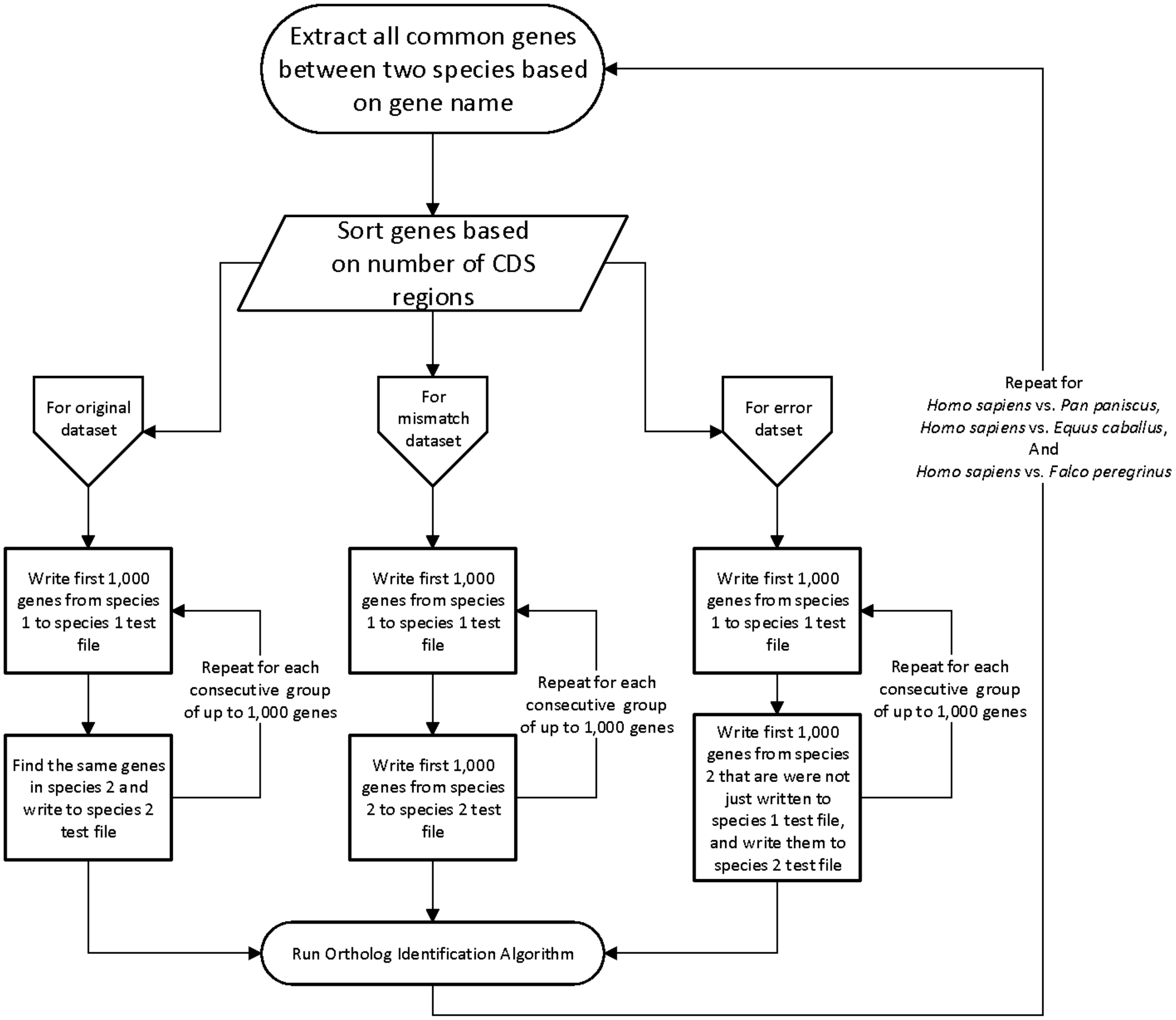

Supplementary Figure 2. Test Set Creation. Three different types of test data sets are created. Original (left) data sets are all true positive orthologs, mismatch data sets (middle) are a random mix of true and false positive orthologs, and error data sets (right) contain no true orthologs. Each data set contains up to 1 000 sequences.

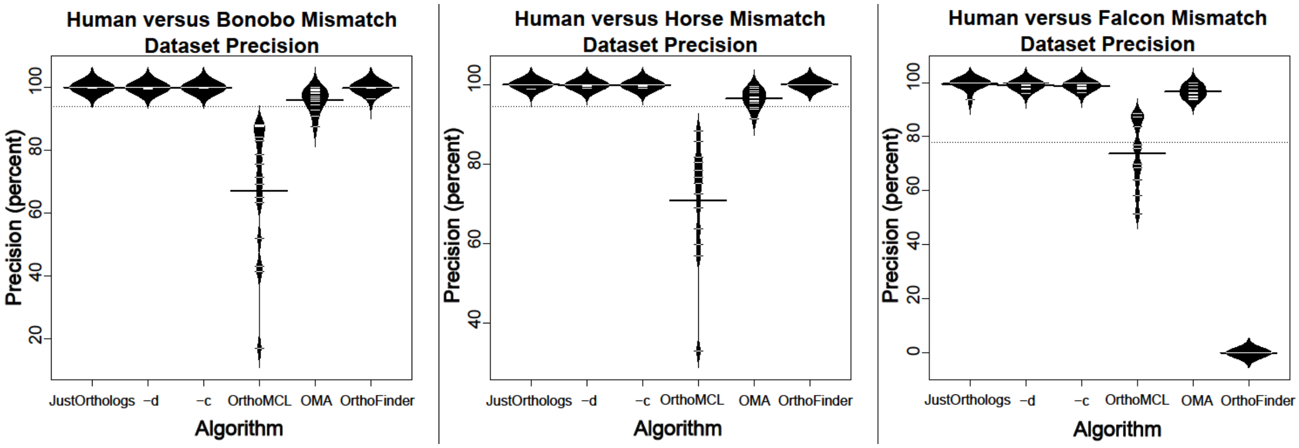

Supplementary Figure 3. Precision Measurements. We present bean plots comparing the precision of the three different settings for JustOrthologs to OrthoMCL, OMA, and OrthoFinder for humans versus bonobos (left), humans versus horse (middle), and humans versus falcon (right). Bean plots display the individual tests as horizontal bars within a shaded density distribution, which allows for easy identification of outliers. The darker horizontal bar shown for each test set is the sample mean, and the dotted line that spans the entire chart is the overall mean from all samples and tests (Kampstra, 2008). Results in this figure are from mismatch test data sets (a mix of real and not real orthologs), which are the best approximation of a real data set. The x-axis labels refer to the algorithm used. From left to right, the algorithms used are: JustOrthologs for closely related species, JustOrthologs for distantly related species, JustOrthologs combined approach, OrthoMCL, OMA, and OrthoFinder.

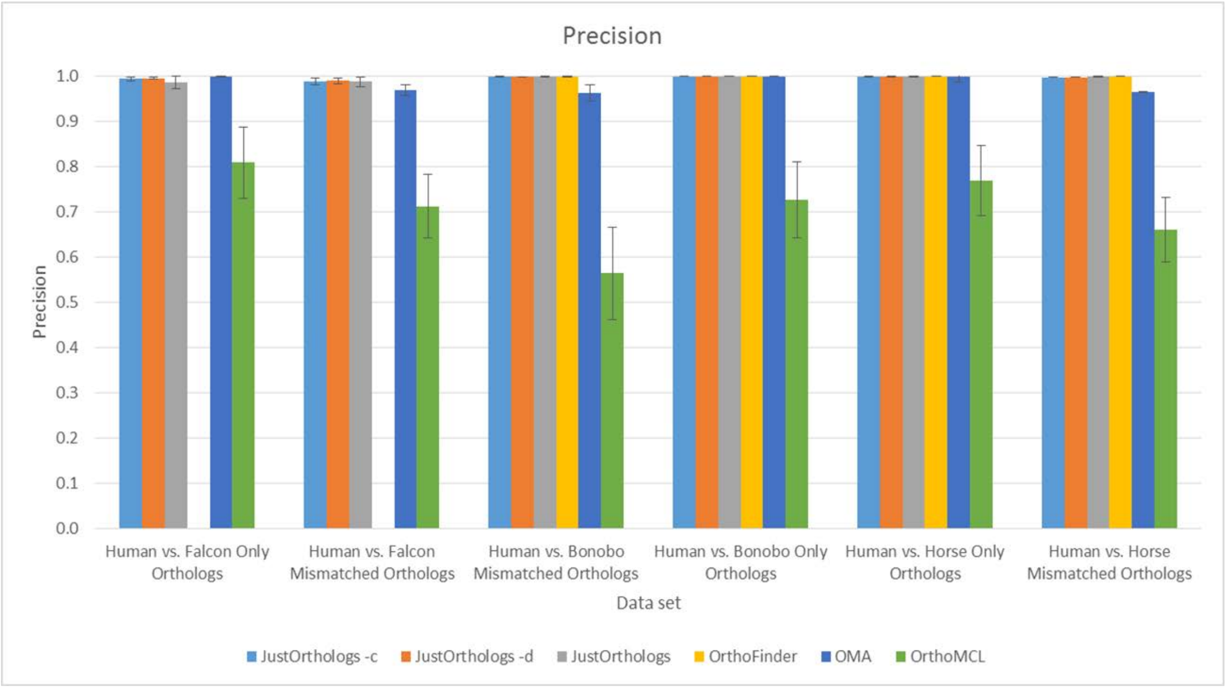

Supplementary Figure 4. Precision for all datasets

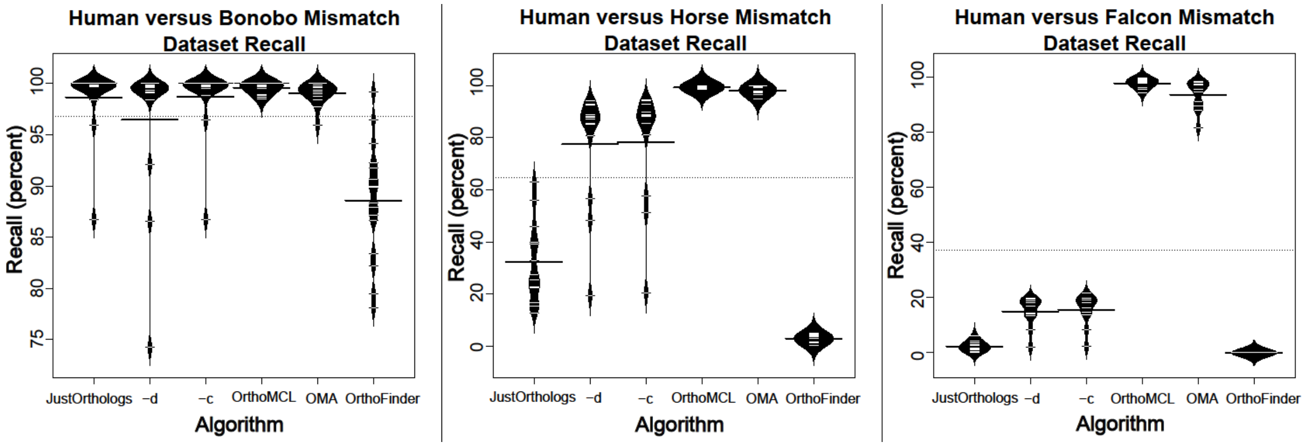

Supplementary Figure 5. Recall Measurements. We present bean plots comparing the recall of the three different settings for JustOrthologs to OrthoMCL, OMA, and OrthoFinder for humans versus bonobos (left), humans versus horse (middle), and humans versus falcon (right). Results are from mismatch test data sets (a mix of real and not real orthologs), which are the best approximation of a real data set. Bean plots and the x-axis labels are as described in Fig. 3.

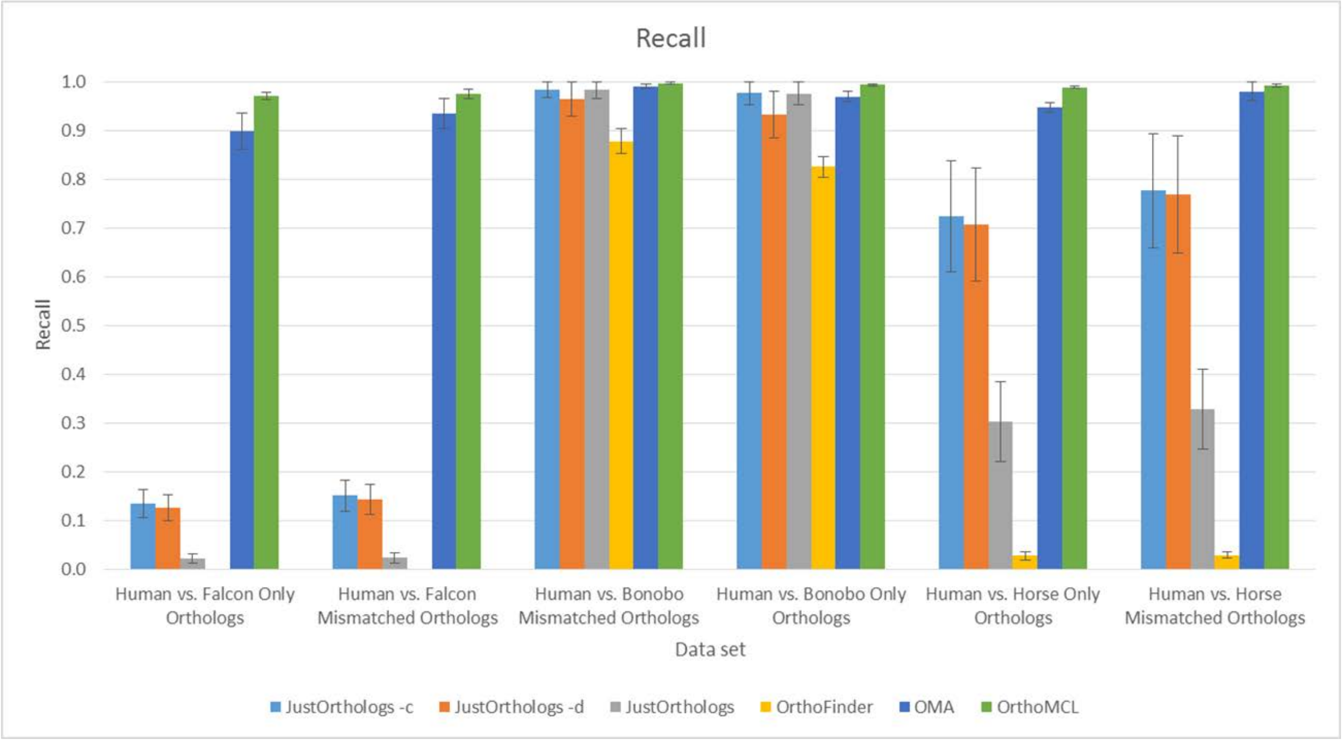

Supplementary Figure 6. Recall for all datasets

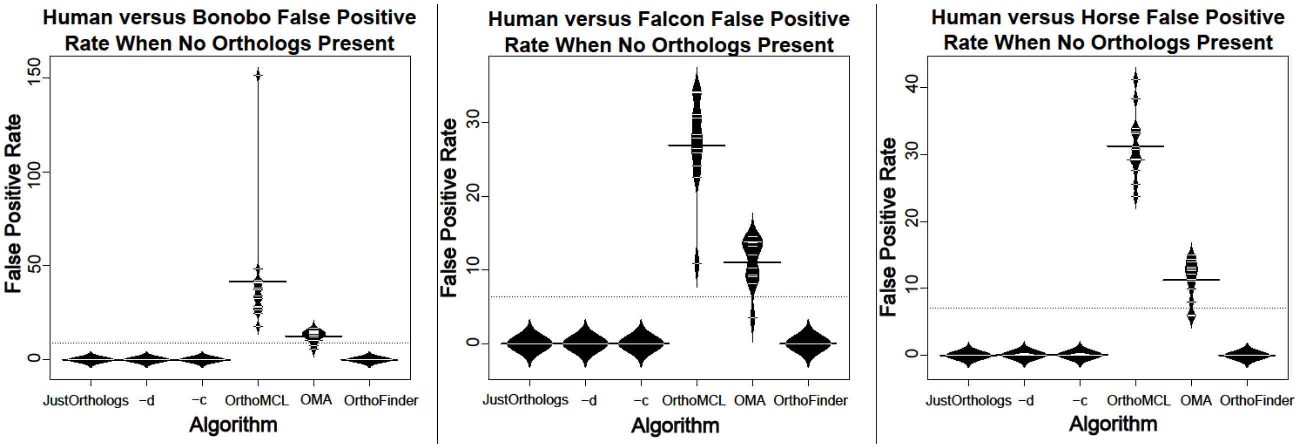

Supplementary Figure 7. False Positive Rate. We present bean plots comparing the false positive rate rate of the three different settings for JustOrthologs, OrthoMCL, OMA, and OrthoFinder for humans versus bonobos (left), humans versus horse (middle), and humans versus falcon (right). Results are from the error dataset, with no true orthologs. These graphs show how many orthologs are reported by each algorithm when no orthologs are present. Bean plots and the x-axis labels are as described in Fig. 3.

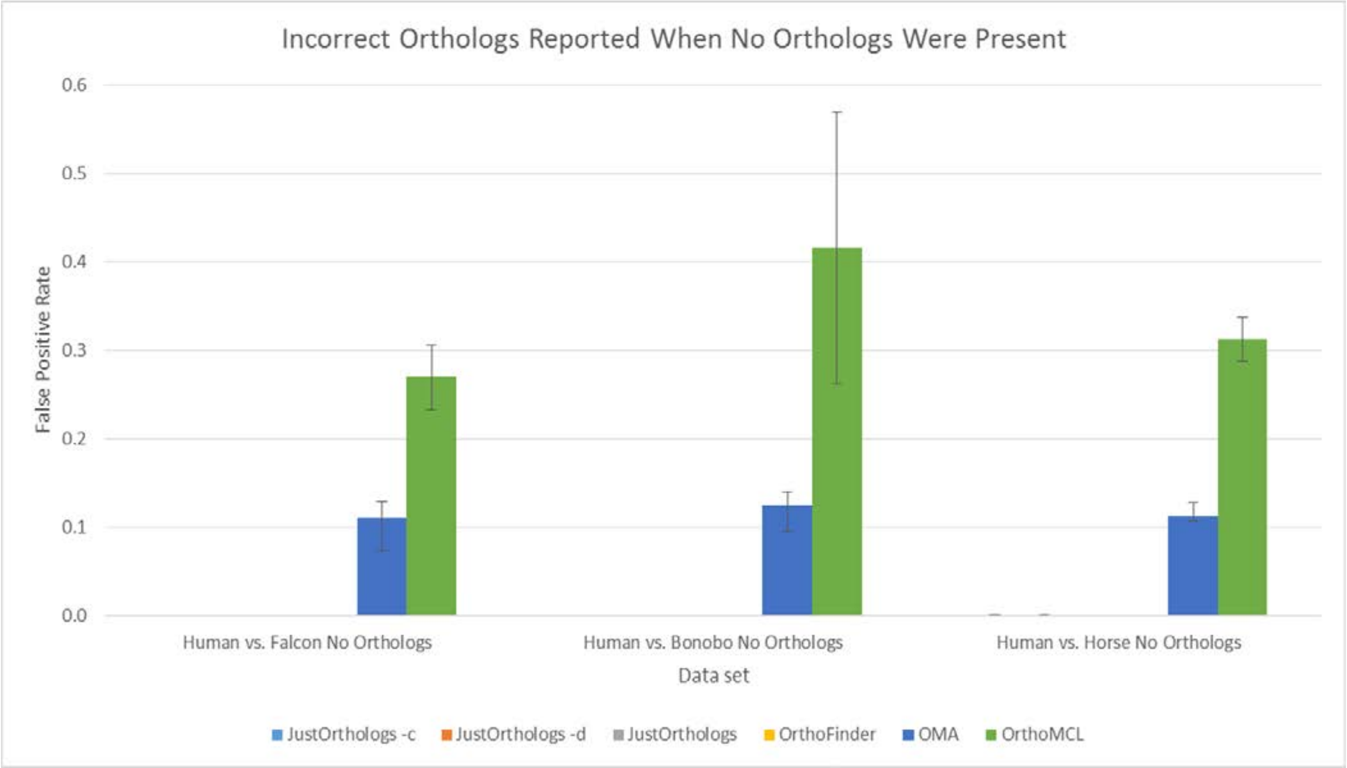

Supplementary Figure 8. False Positive Rate for all datasets

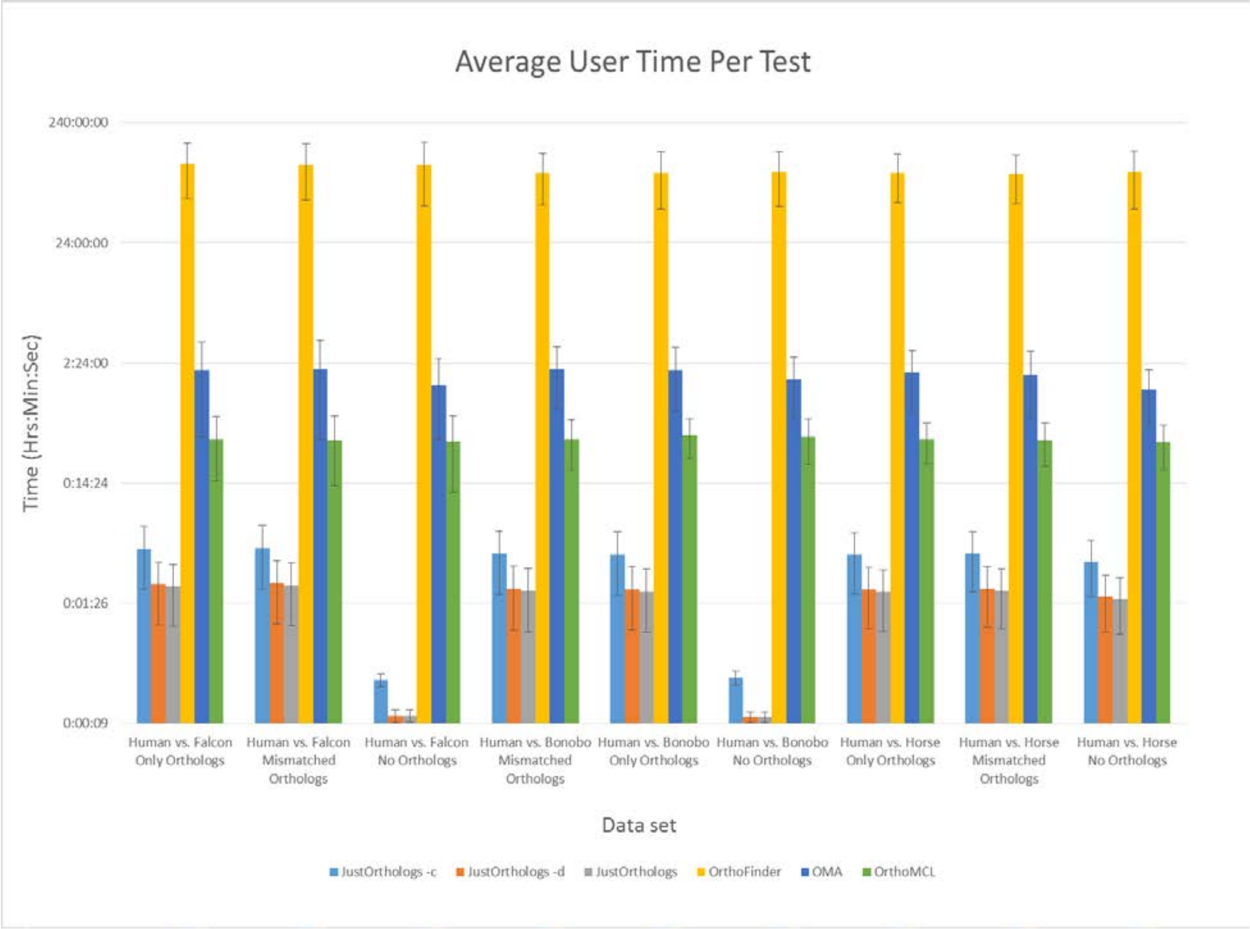

Supplementary Figure 9. User Time for all datasets

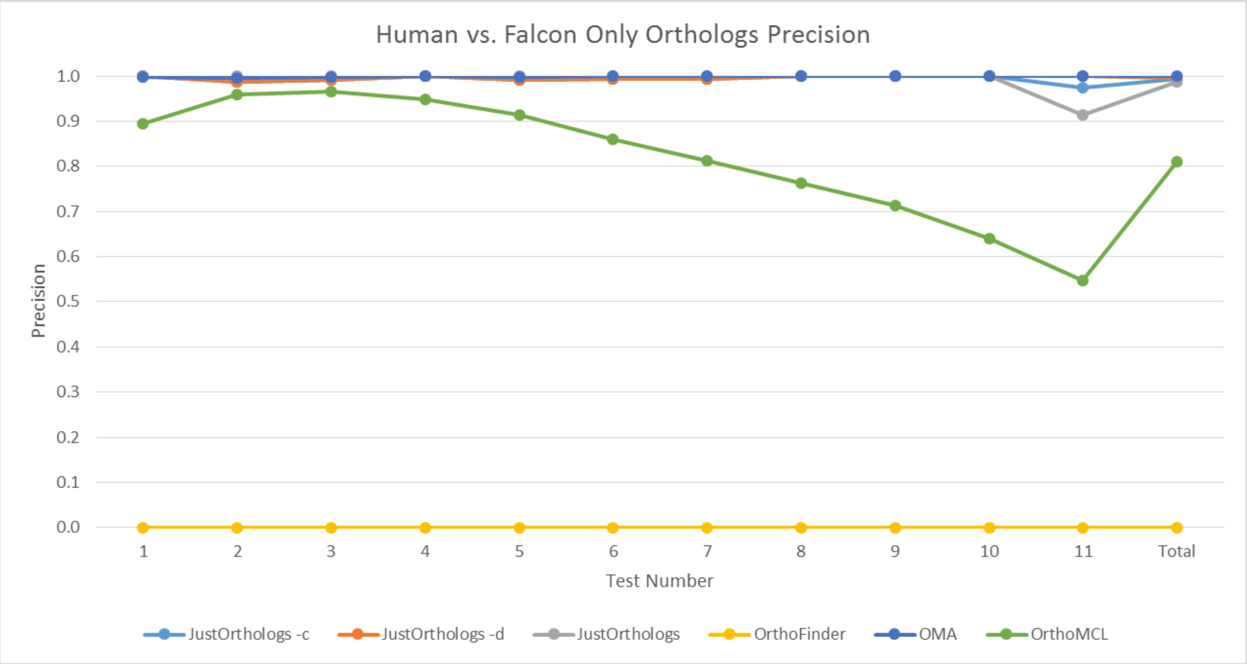

Supplementary Figure 10. Precision for humans versus falcons for each test case where only orthologs are present

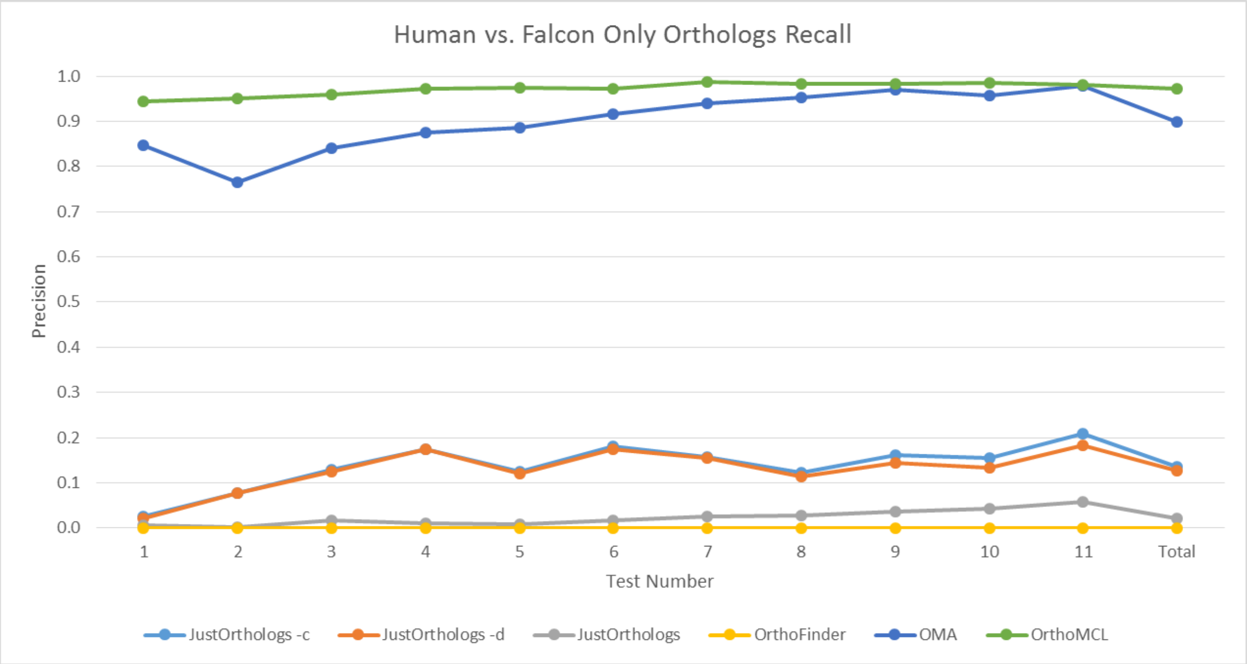

Supplementary Figure 11. Recall for humans versus falcons for each test case where only orthologs are present

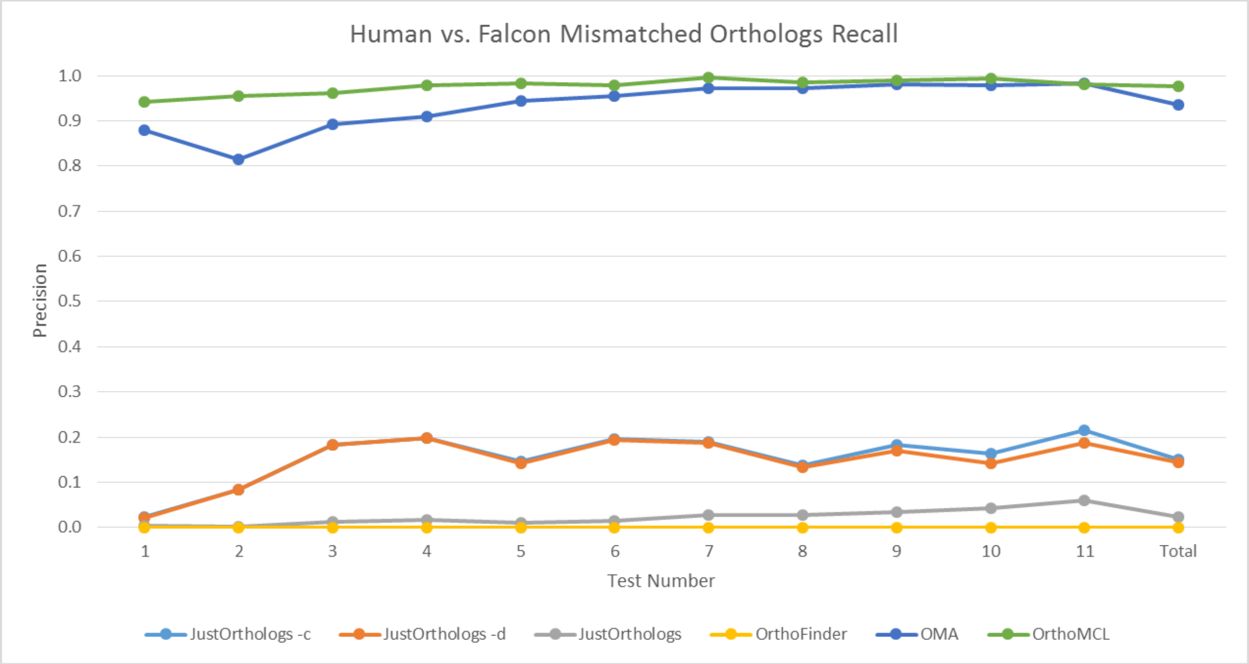

Supplementary Figure 12. Recall for humans versus falcons for each test case where some orthologs are present

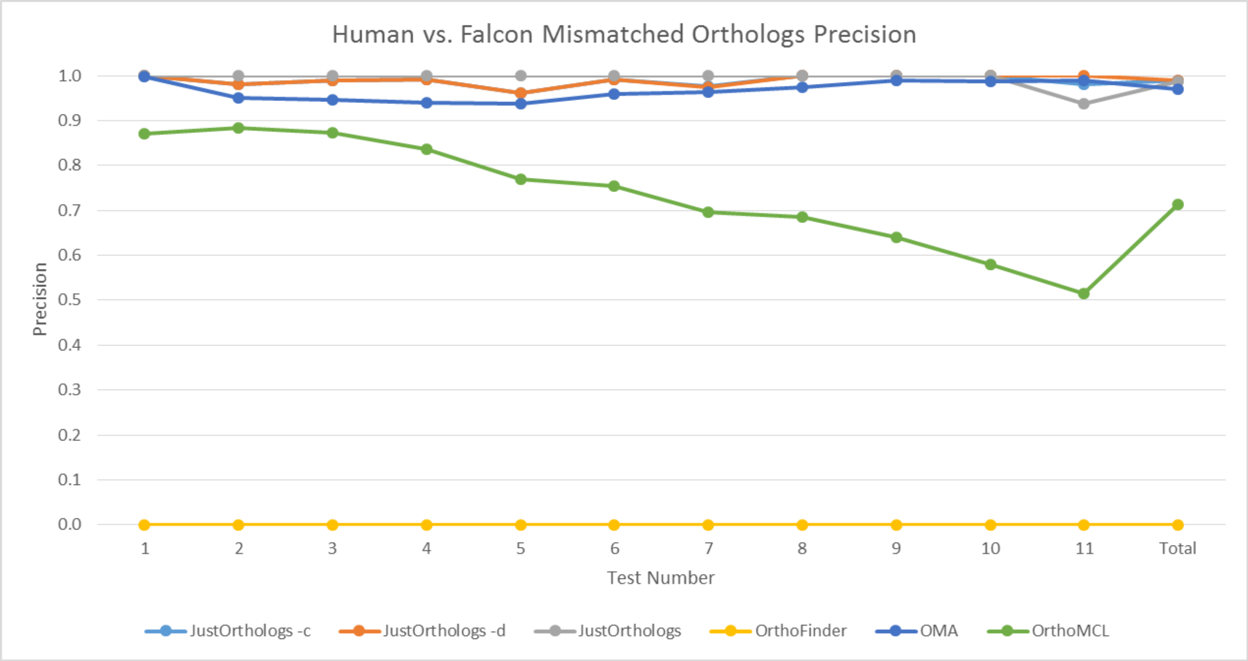

Supplementary Figure 13. Precision for humans versus falcons for each test case where some orthologs are present

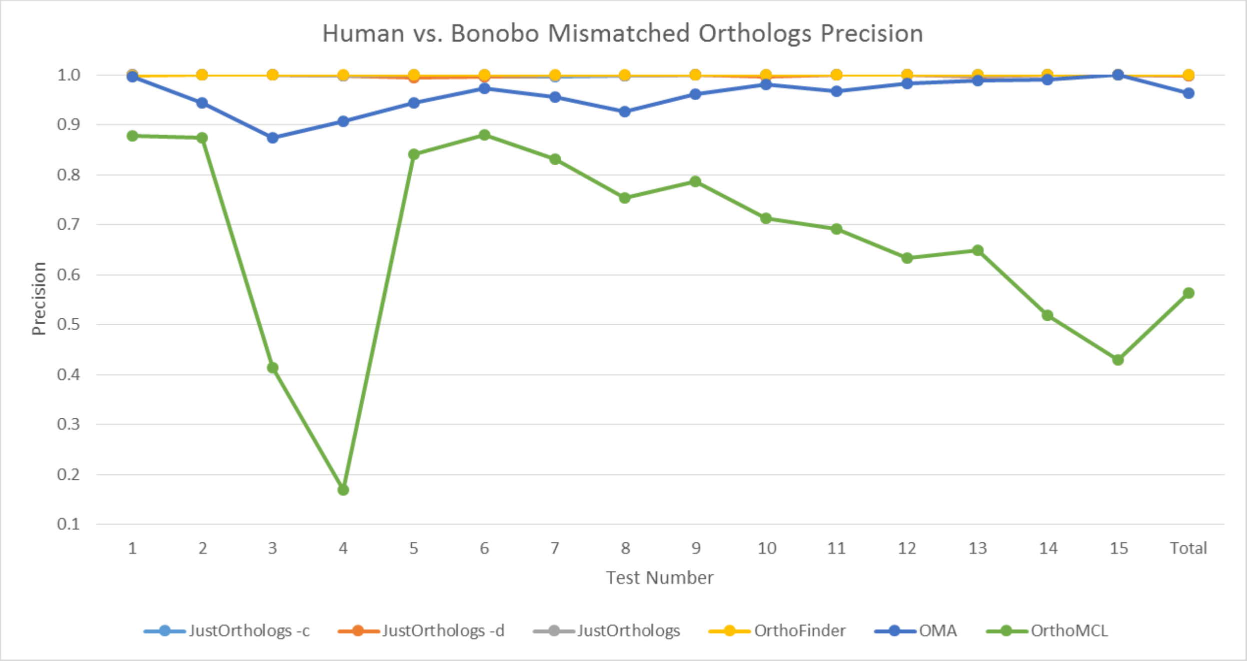

Supplementary Figure 14. Precision for humans versus bonobo for each test case where some orthologs are present

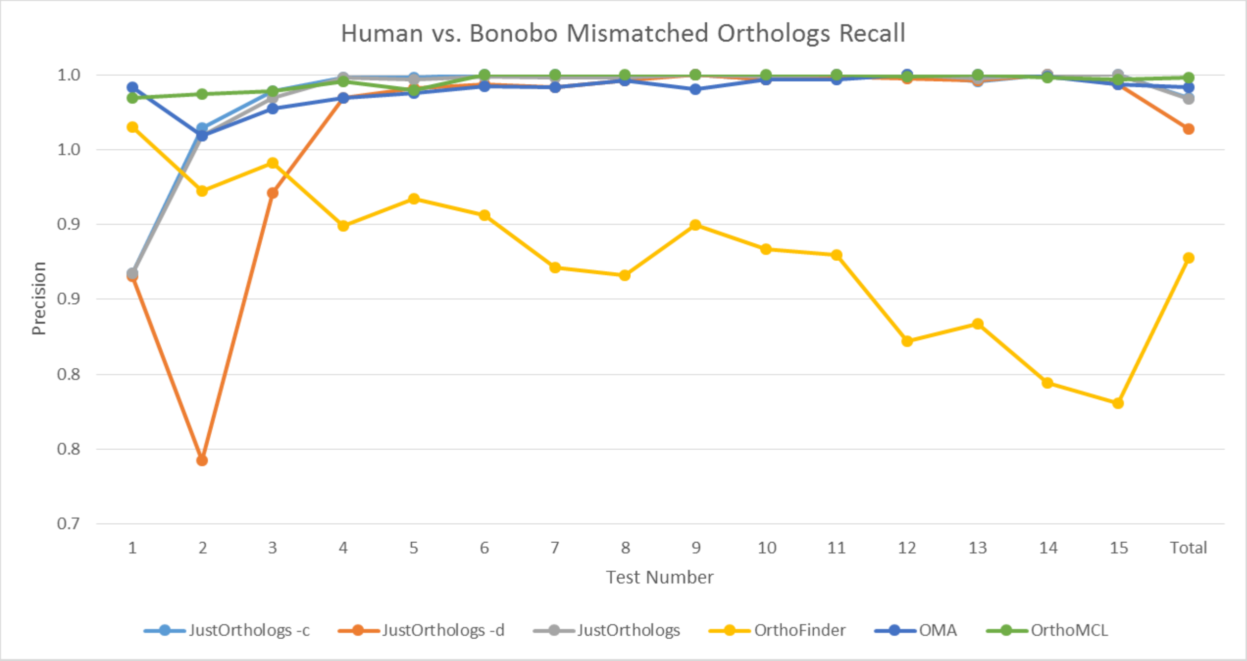

Supplementary Figure 15. Recall for humans versus bonobo for each test case where some orthologs are present

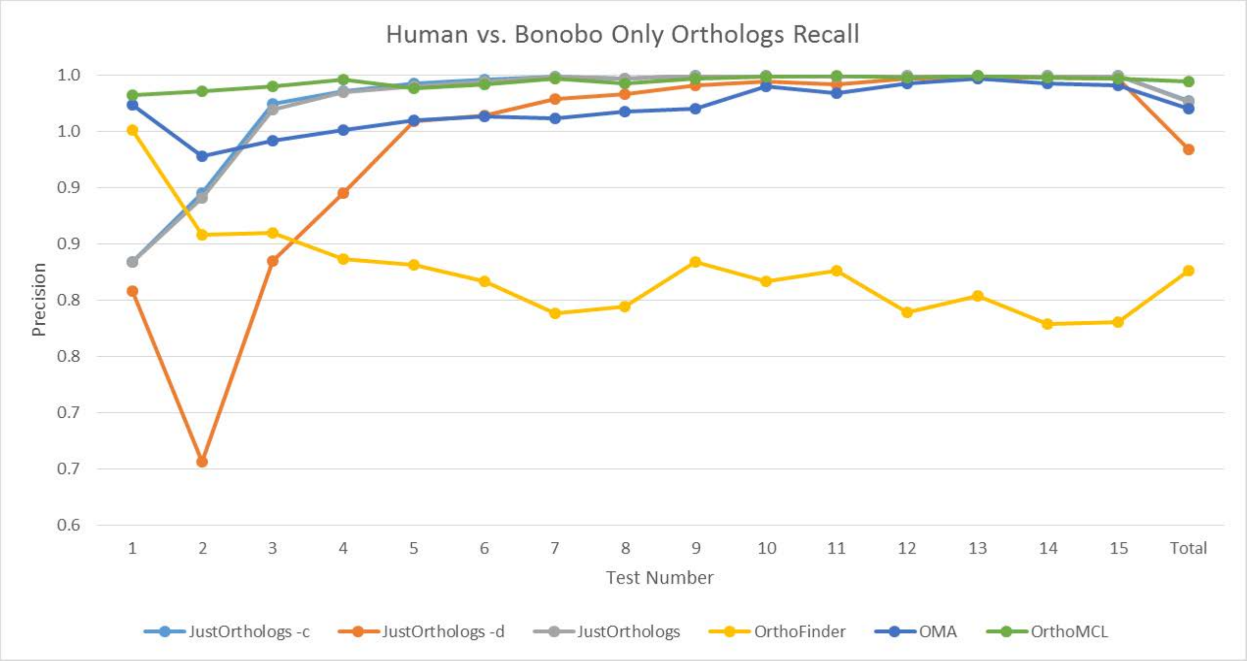

Supplementary Figure 16. Recall for humans versus bonobo for each test case where only orthologs are present

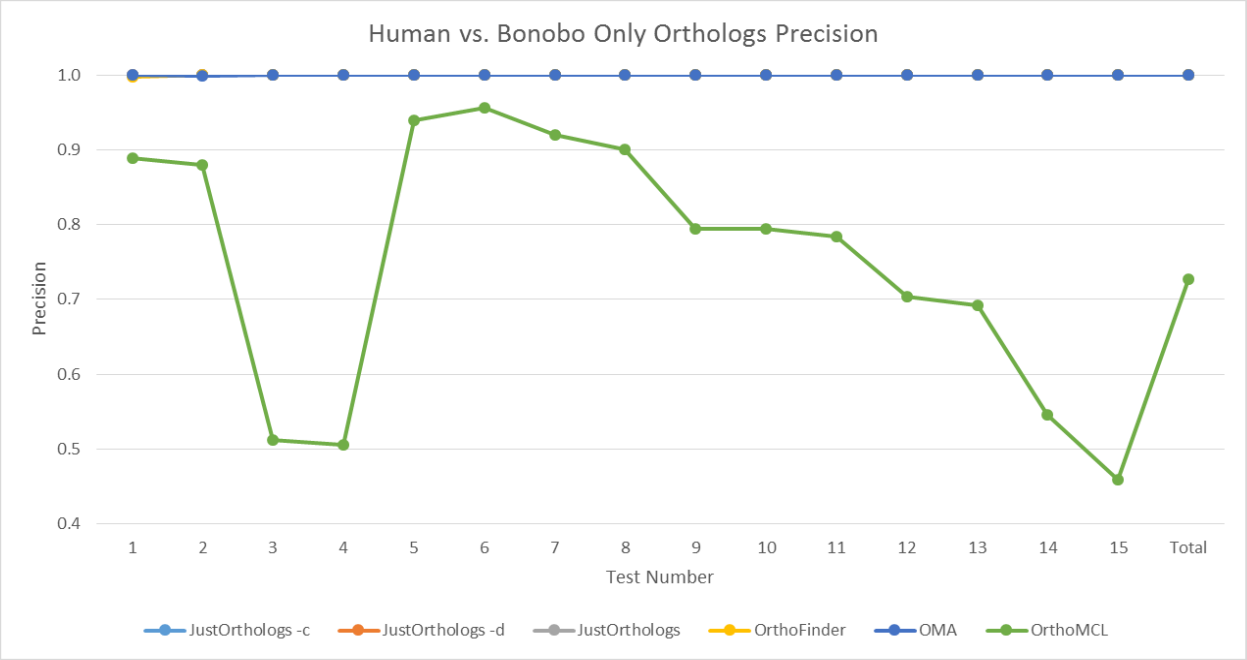

Supplementary Figure 17. Precision for humans versus bonobo for each test case where only orthologs are present

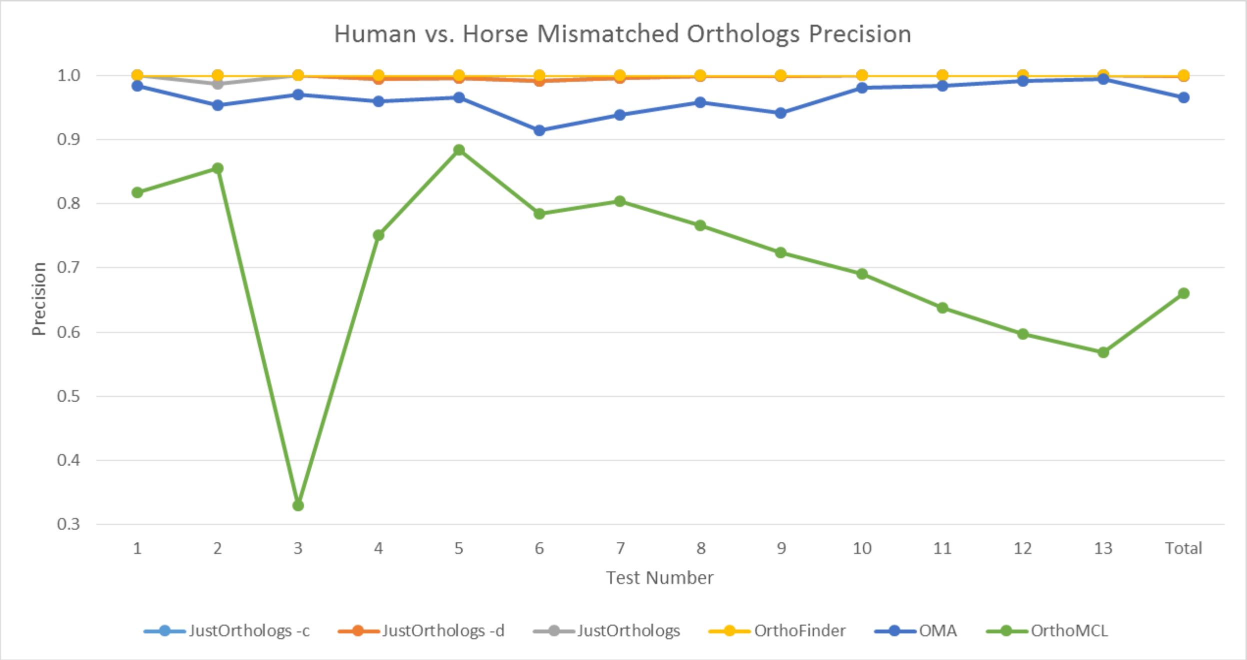

Supplementary Figure 18. Precision for humans versus horse for each test case where only orthologs are present

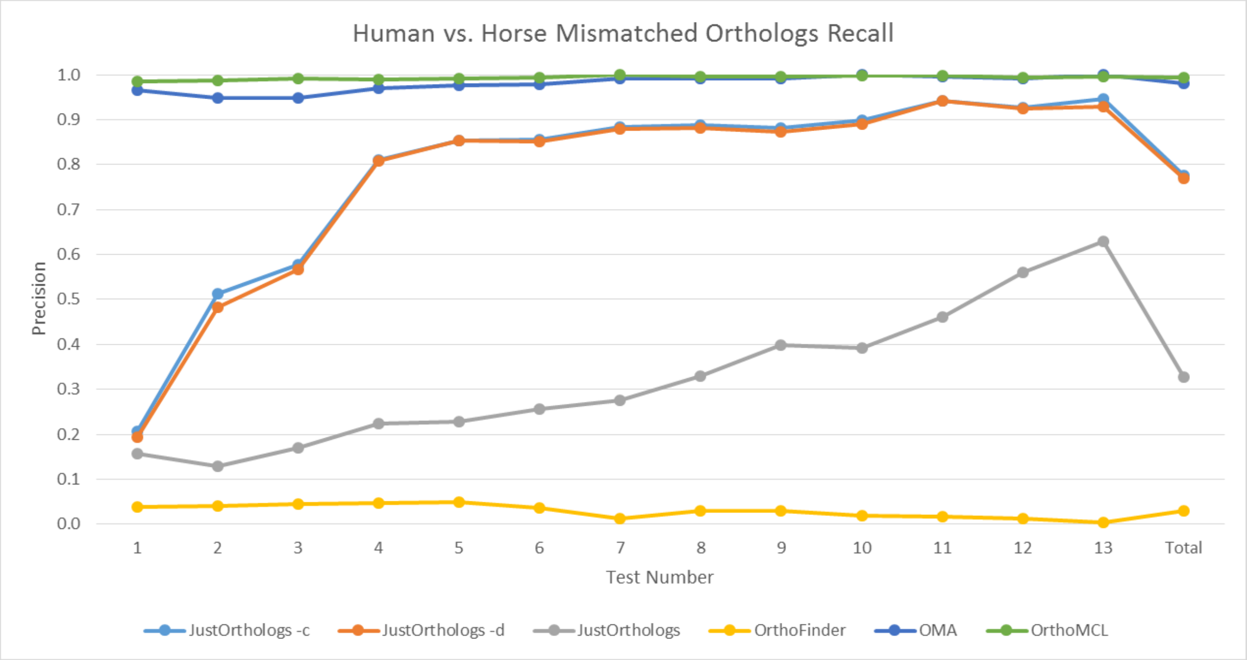

Supplementary Figure 19. Recall for humans versus horse for each test case where only orthologs are present

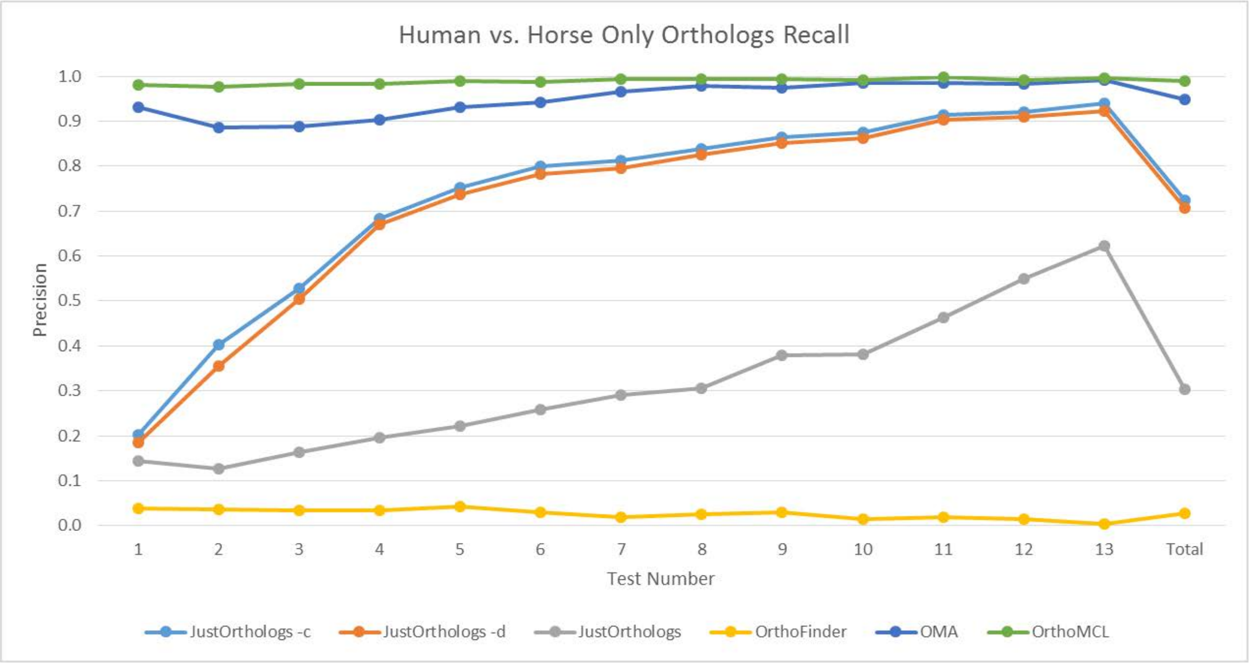

Supplementary Figure 20. Recall for humans versus horse for each test case where only orthologs are present

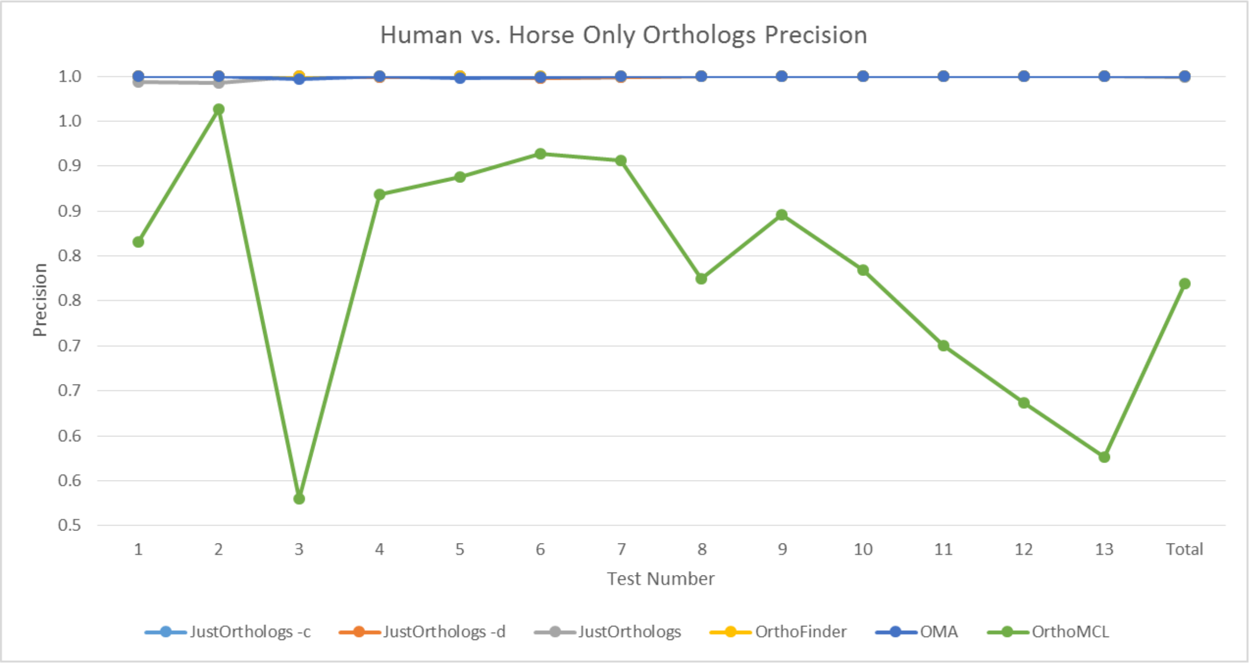

Supplementary Figure 21. Precision for humans versus horse for each test case where only orthologs are present

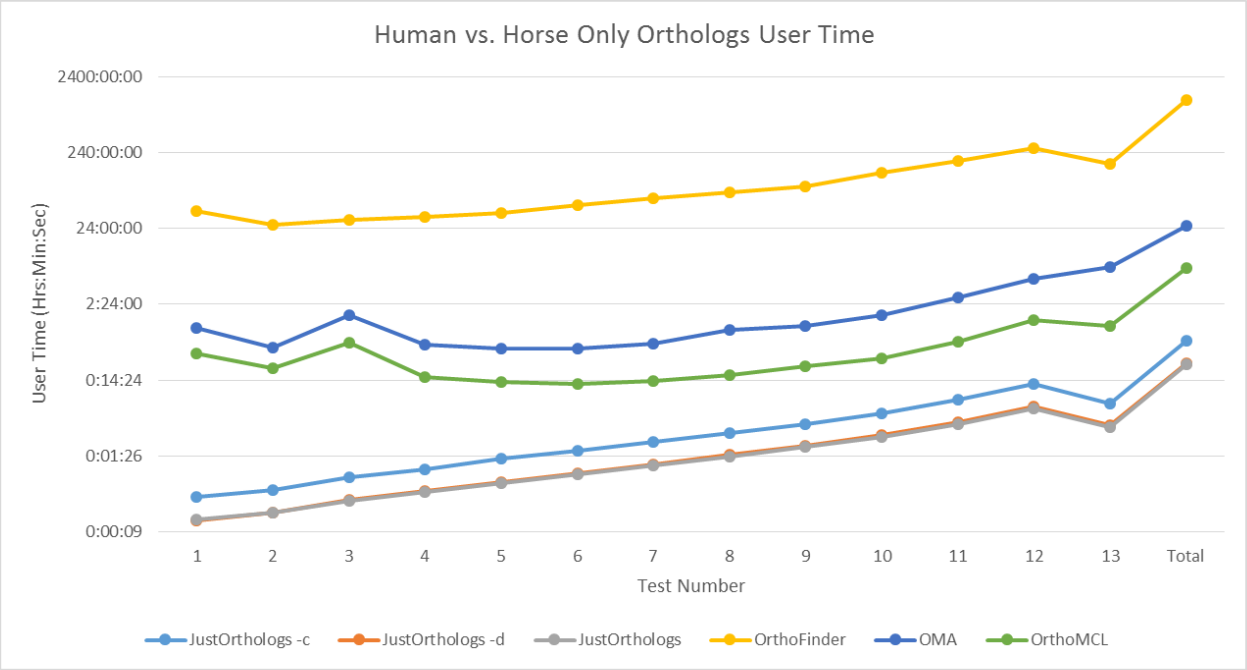

Supplementary Figure 22. User time for humans versus horse for each test case where only orthologs are present

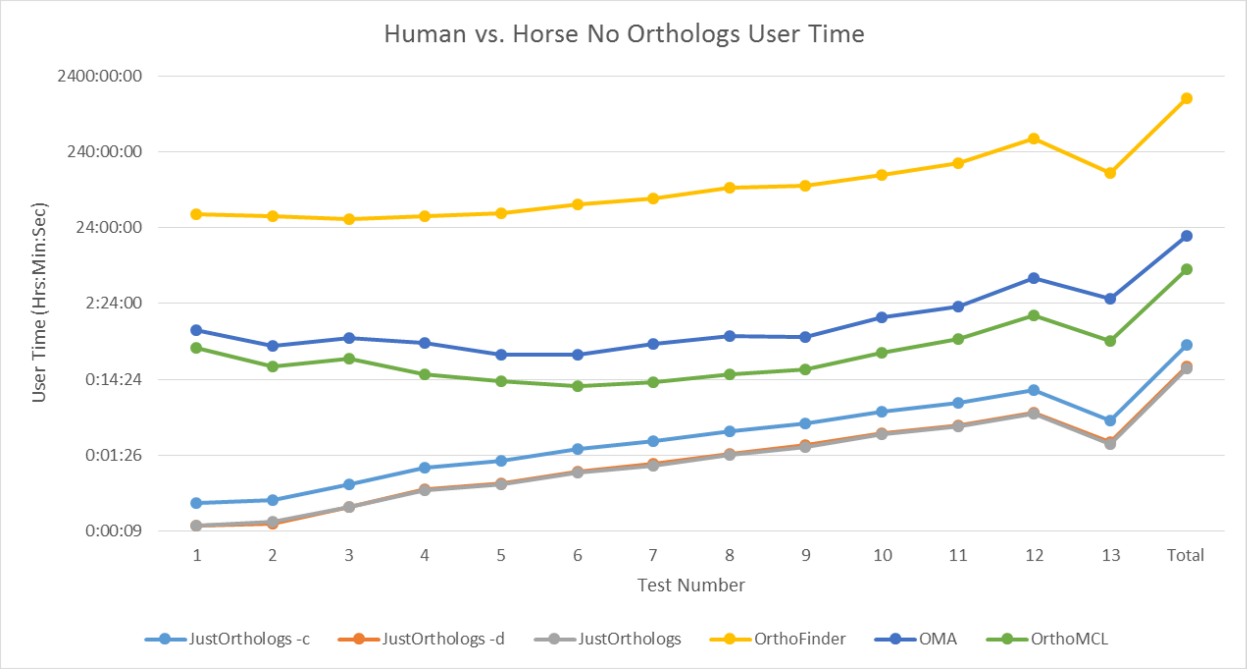

Supplementary Figure 23. User time for humans versus horse for each test case where no orthologs are present

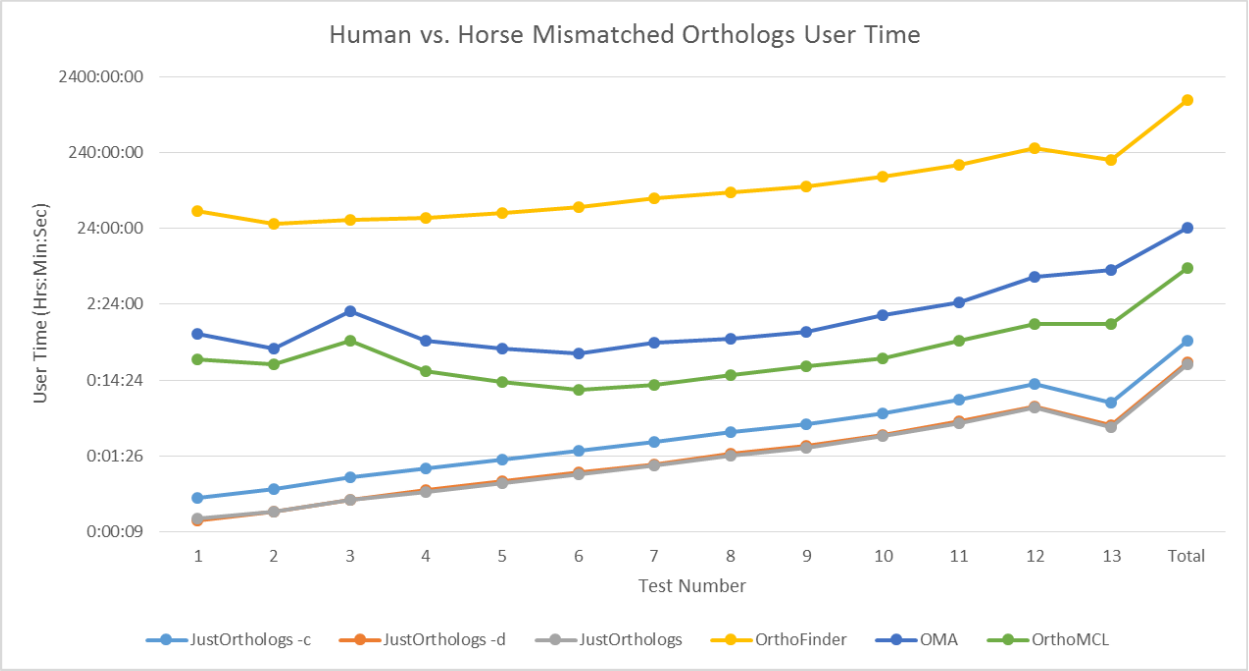

Supplementary Figure 24. User time for humans versus horse for each test case where some orthologs are present

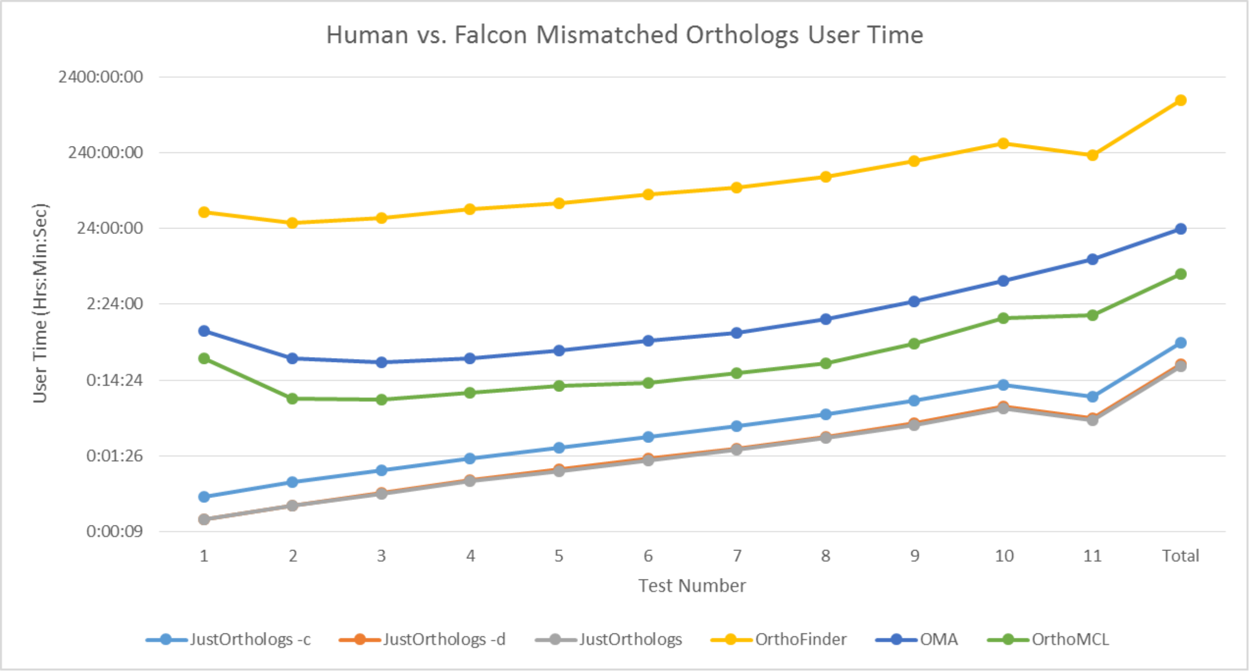

Supplementary Figure 25. User time for humans versus falcon for each test case where some orthologs are present

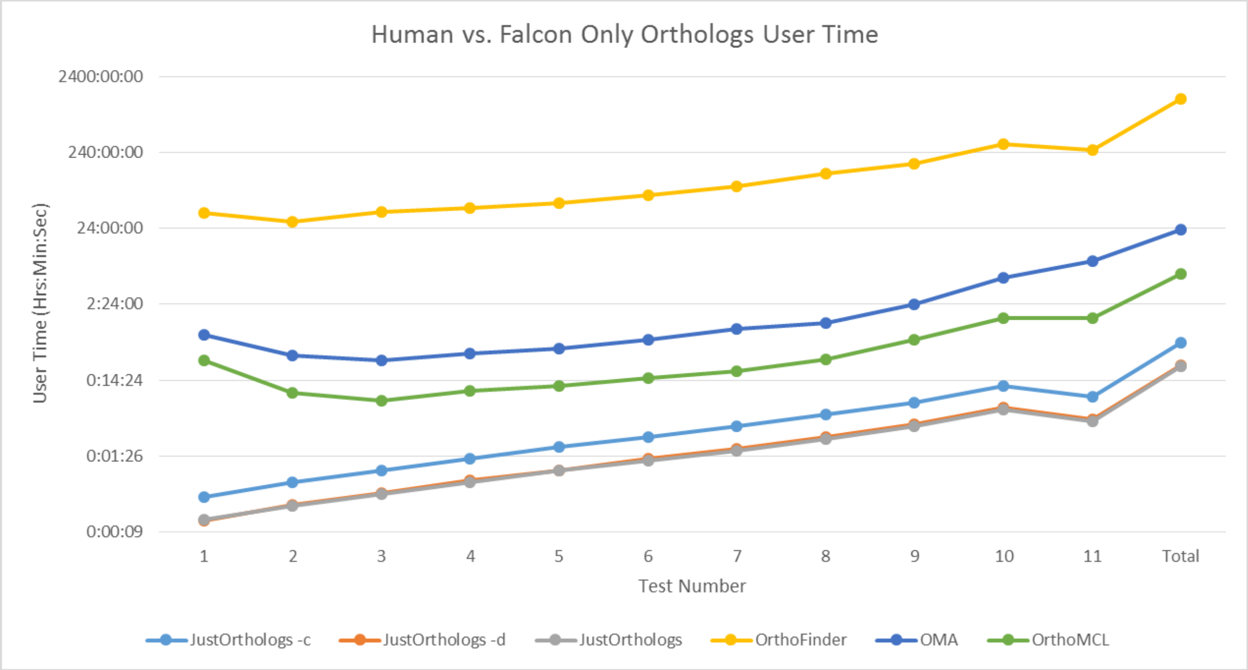

Supplementary Figure 26. User time for humans versus falcon for each test case where only orthologs are present

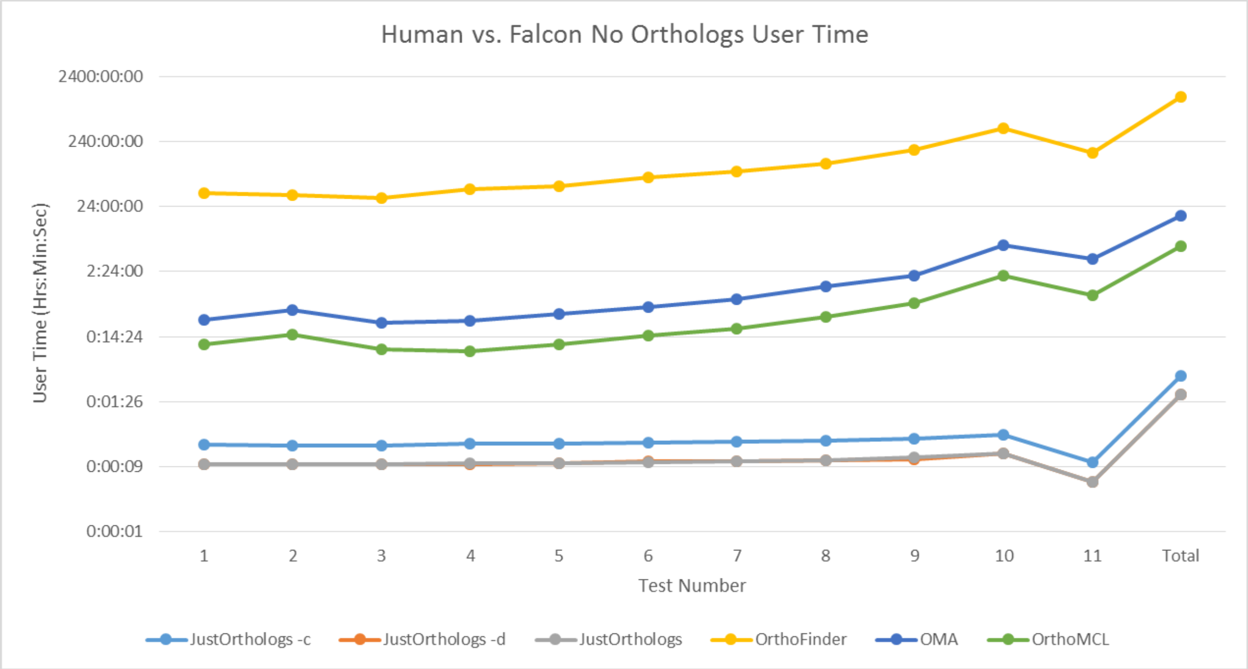

Supplementary Figure 27. User time for humans versus falcon for each test case where no orthologs are present

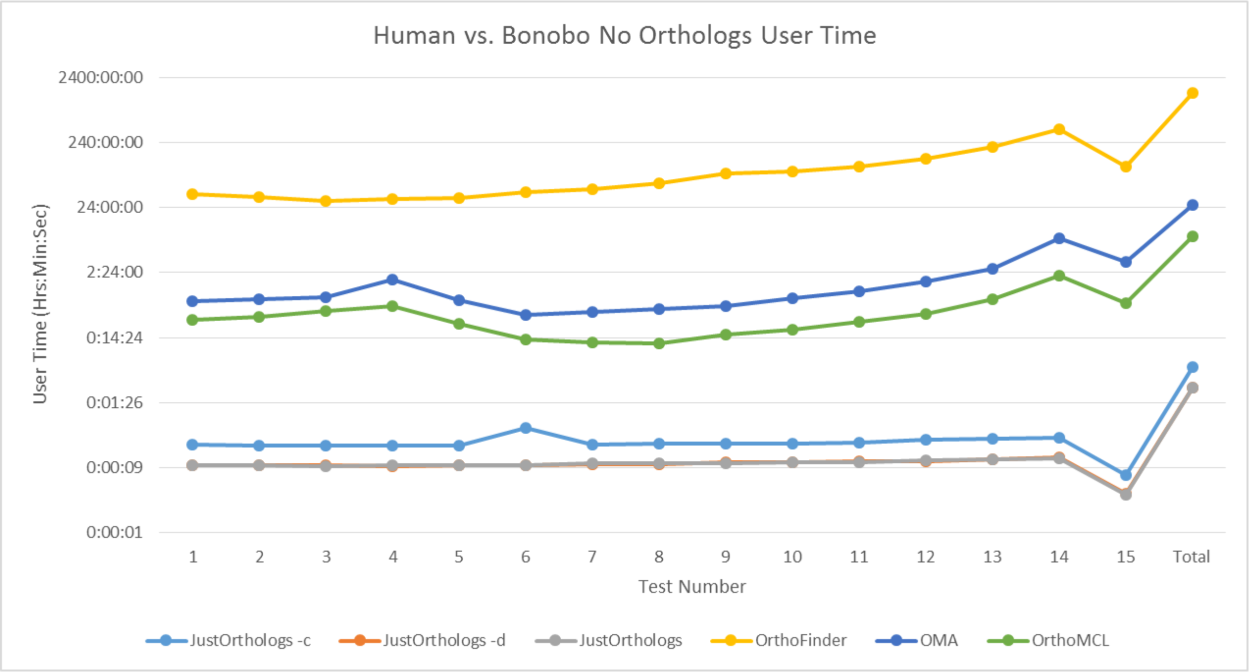

Supplementary Figure 28. User time for humans versus bonobo for each test case where no orthologs are present

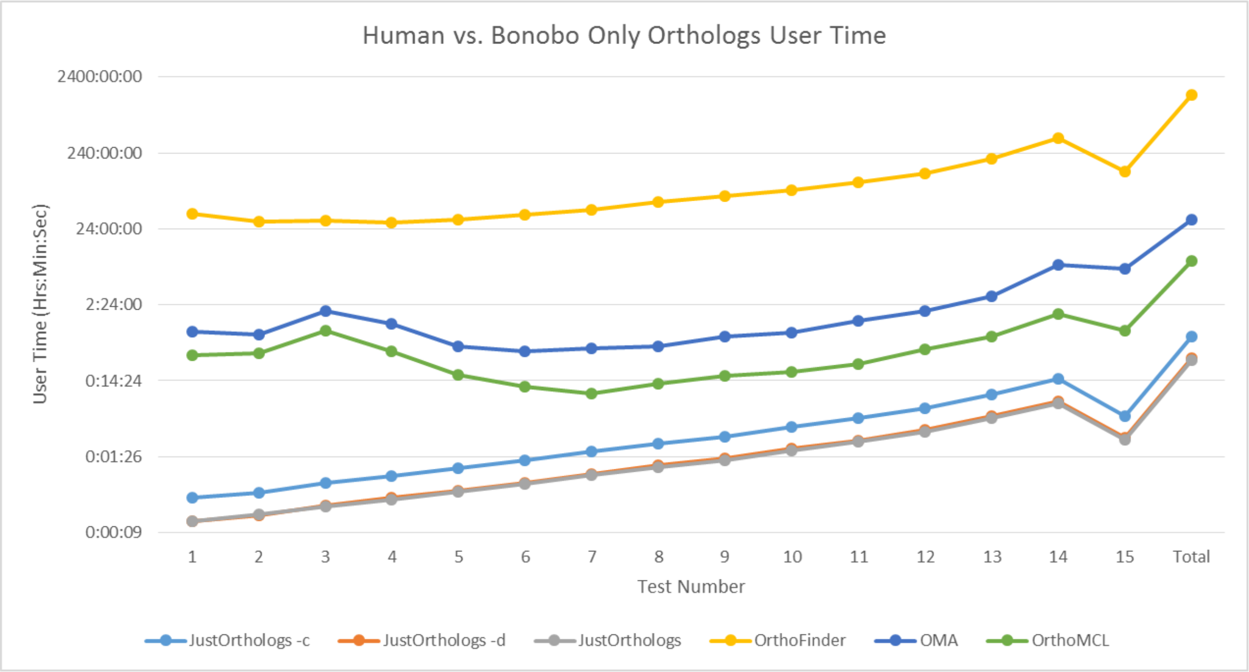

Supplementary Figure 29. User time for humans versus bonobo for each test case where only orthologs are present

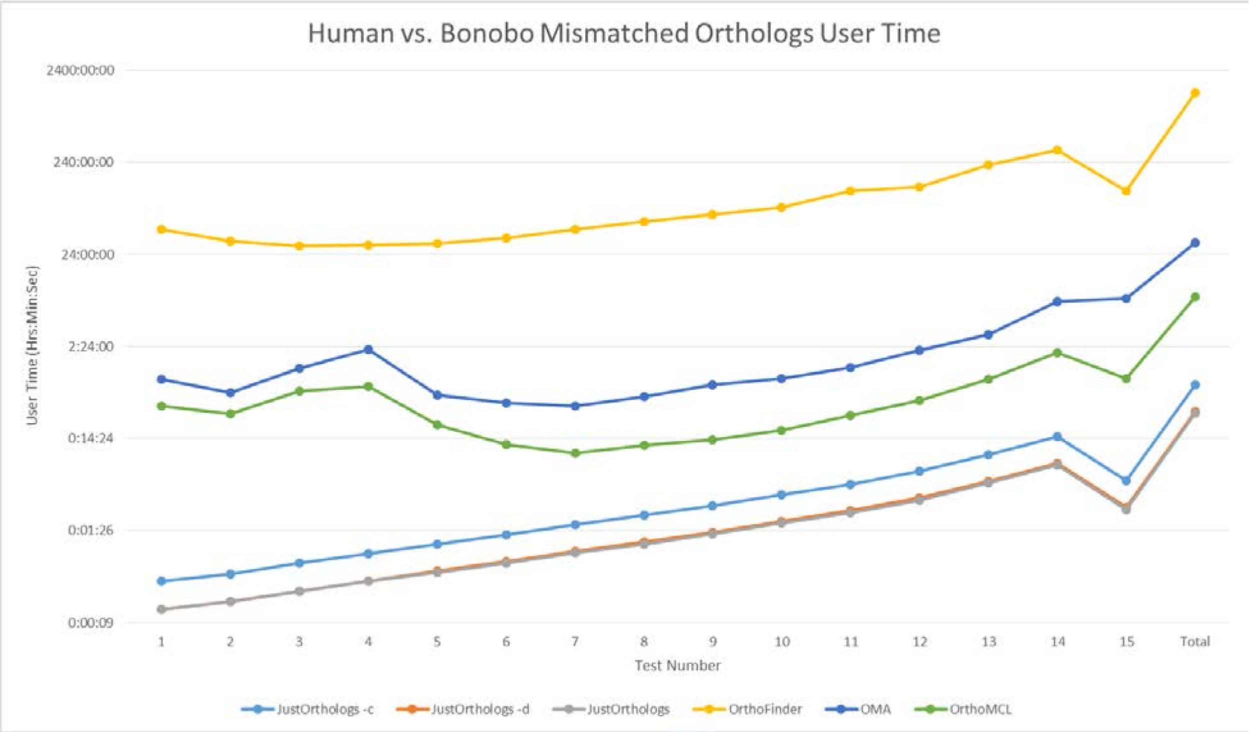

Supplementary Figure 30. User time for humans versus bonobo for each test case where some orthologs are present

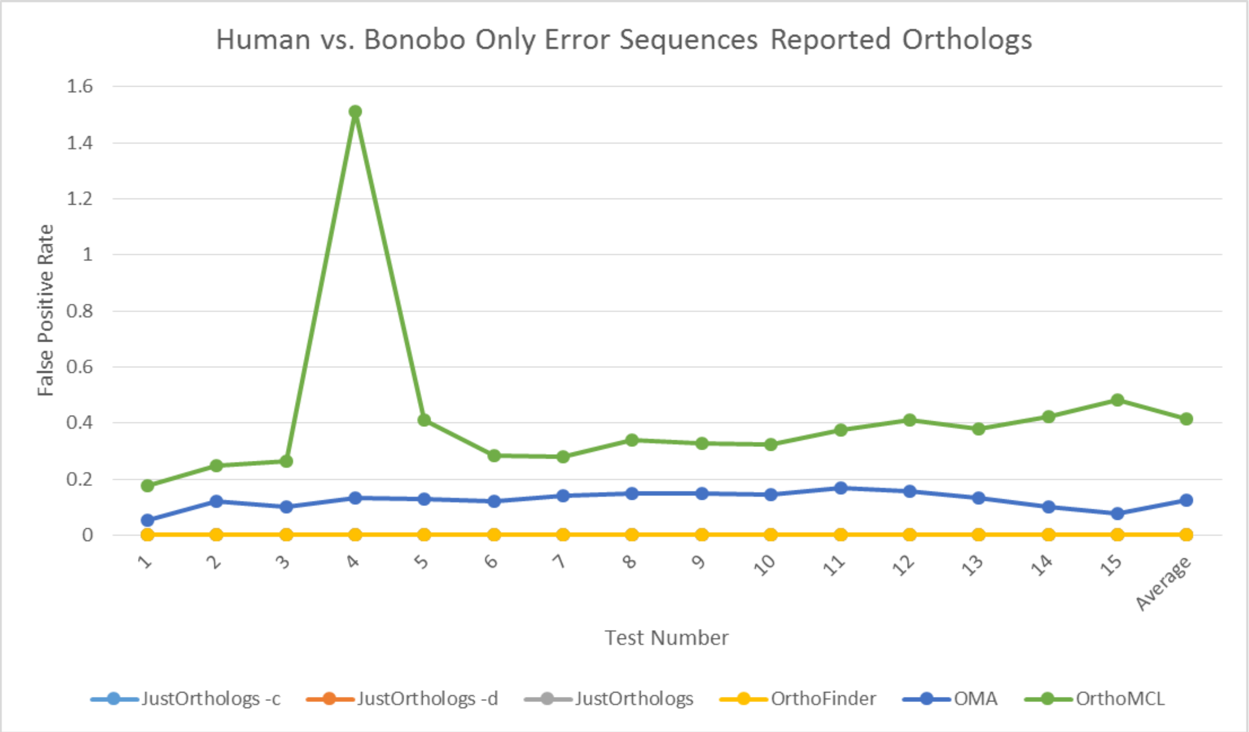

Supplementary Figure 31. False positive orthologs reported for humans versus bonobo for each test case where no orthologs are present

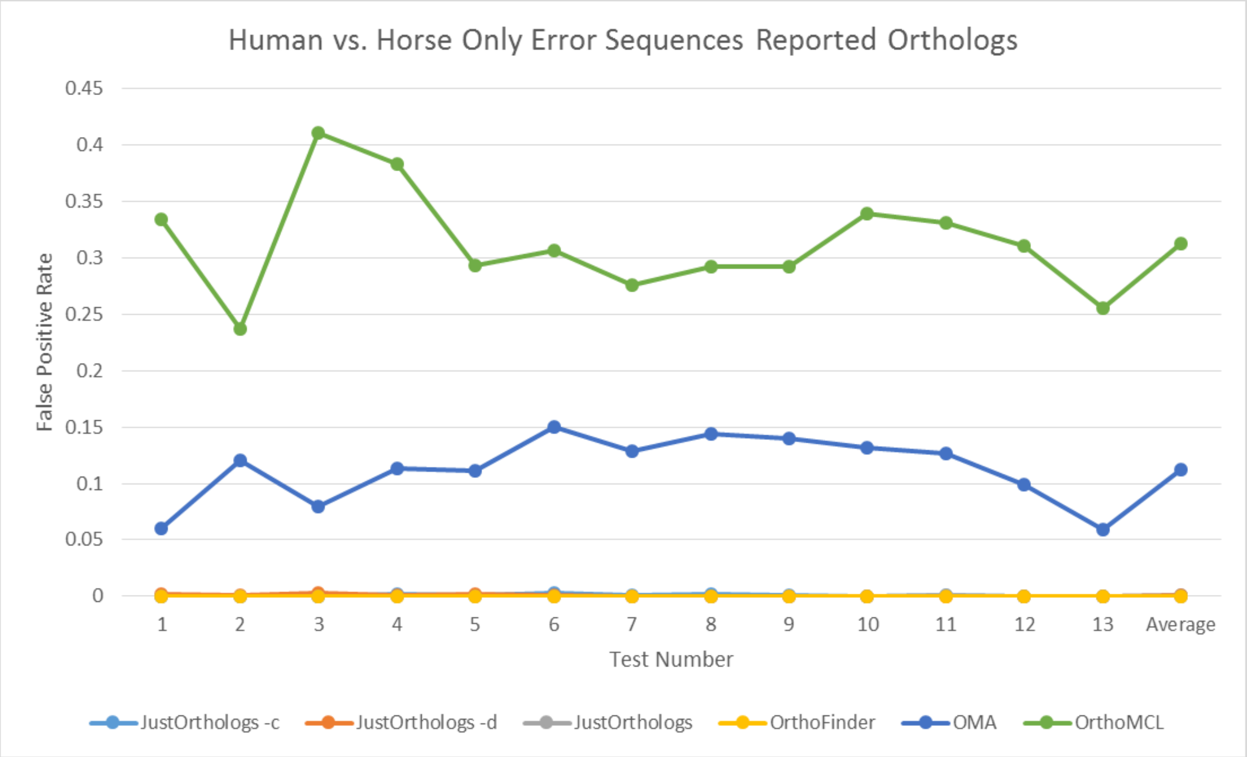

Supplementary Figure 32. False positive orthologs reported for humans versus horse for each test case where no orthologs are present

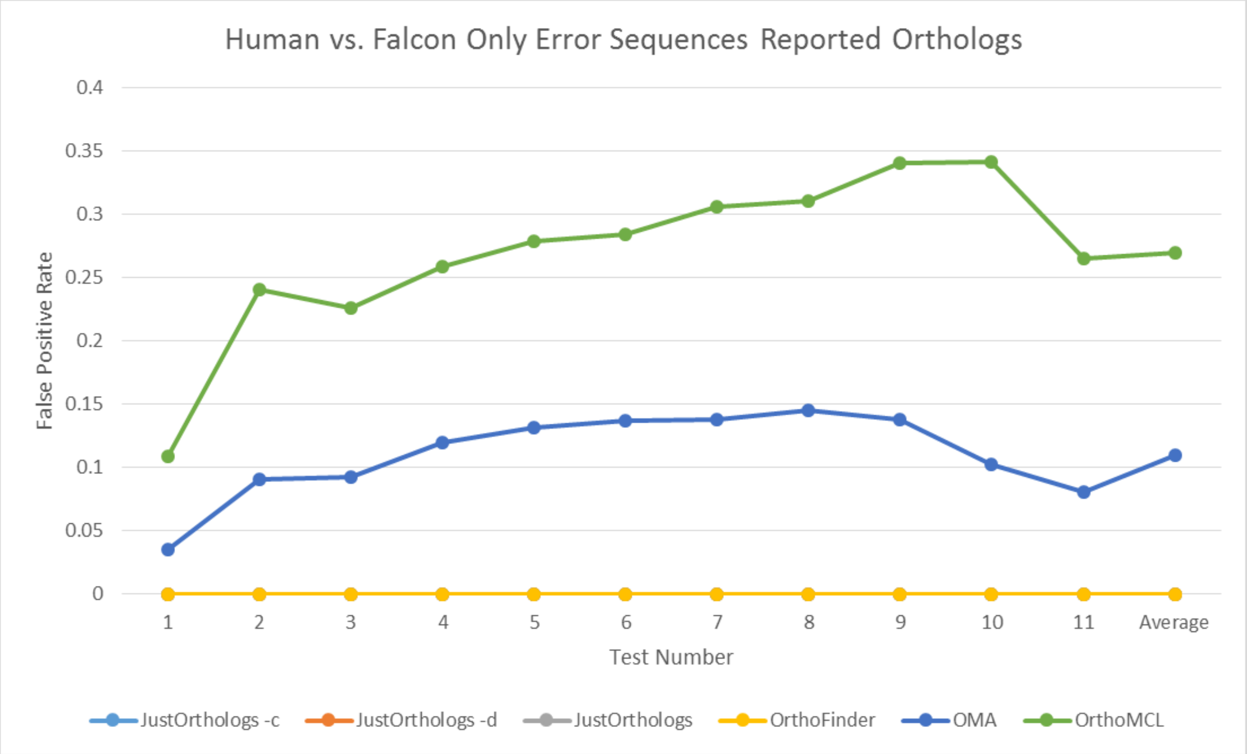

Supplementary Figure 33. False positive orthologs reported for humans versus falcon for each test case where no orthologs are present
